# Designed Metal-Containing Peptoid Membranes as Enzyme Mimetics for Catalytic Organophosphate Degradation

**DOI:** 10.1021/acsami.3c11816

**Published:** 2023-10-25

**Authors:** Thi Kim Hoang Trinh, Tengyue Jian, Biao Jin, Dan-Thien Nguyen, Ronald N. Zuckermann, Chun-Long Chen

**Affiliations:** † Physical Sciences Division, Pacific Northwest National Laboratory, Richland, Washington 99352, United States; ^‡^Molecular Foundry, Lawrence Berkeley National Laboratory, 1 Cyclotron Rd., Berkeley, California 94720, United States; ∥ Department of Chemical Engineering, University of Washington, Seattle, Washington 98195, United States

**Keywords:** peptoid, self-assembly, 2D crystalline membrane, enzyme mimic, organophosphate

## Abstract

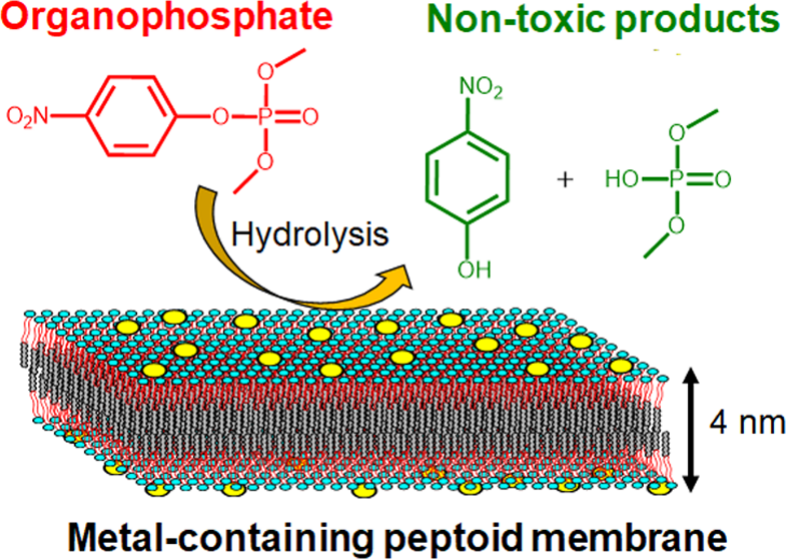

The detoxification of lethal organophosphate (OP) residues in the environment is crucial to prevent human exposure and protect modern society. Despite serving as excellent catalysts for OP degradation, natural enzymes require costly preparation and readily deactivate upon exposure to environmental conditions. Herein, we designed and prepared a series of phosphotriesterase mimics based on stable, self-assembled peptoid membranes to overcome these limitations of the enzymes and effectively catalyze the hydrolysis of dimethyl *p*-nitrophenyl phosphate (DMNP)—a nerve agent simulant. By covalently attaching metal-binding ligands to peptoid N-termini, we attained enzyme mimetics in the form of surface-functionalized crystalline nanomembranes. These nanomembranes display a precisely controlled arrangement of coordinated metal ions, which resemble the active sites found in phosphotriesterases to promote DMNP hydrolysis. Moreover, using these highly programmable peptoid nanomembranes allows for tuning the local chemical environment of the coordinated metal ion to achieve enhanced hydrolysis activity. Among the crystalline membranes that are active for DMNP degradation, those assembled from peptoids containing bis-quinoline ligands with an adjacent phenyl side chain showed the highest hydrolytic activity with a 219-fold rate acceleration over the background, demonstrating the important role of the hydrophobic environment in proximity to the active sites. Furthermore, these membranes exhibited remarkable stability and were able to retain their catalytic activity after heating to 60 °C and after multiple uses. This work provides insights into the principal features to construct a new class of biomimetic materials with high catalytic efficiency, cost-effectiveness, and reusability applied in nerve agent detoxification.

## Introduction

**Figure 1:**
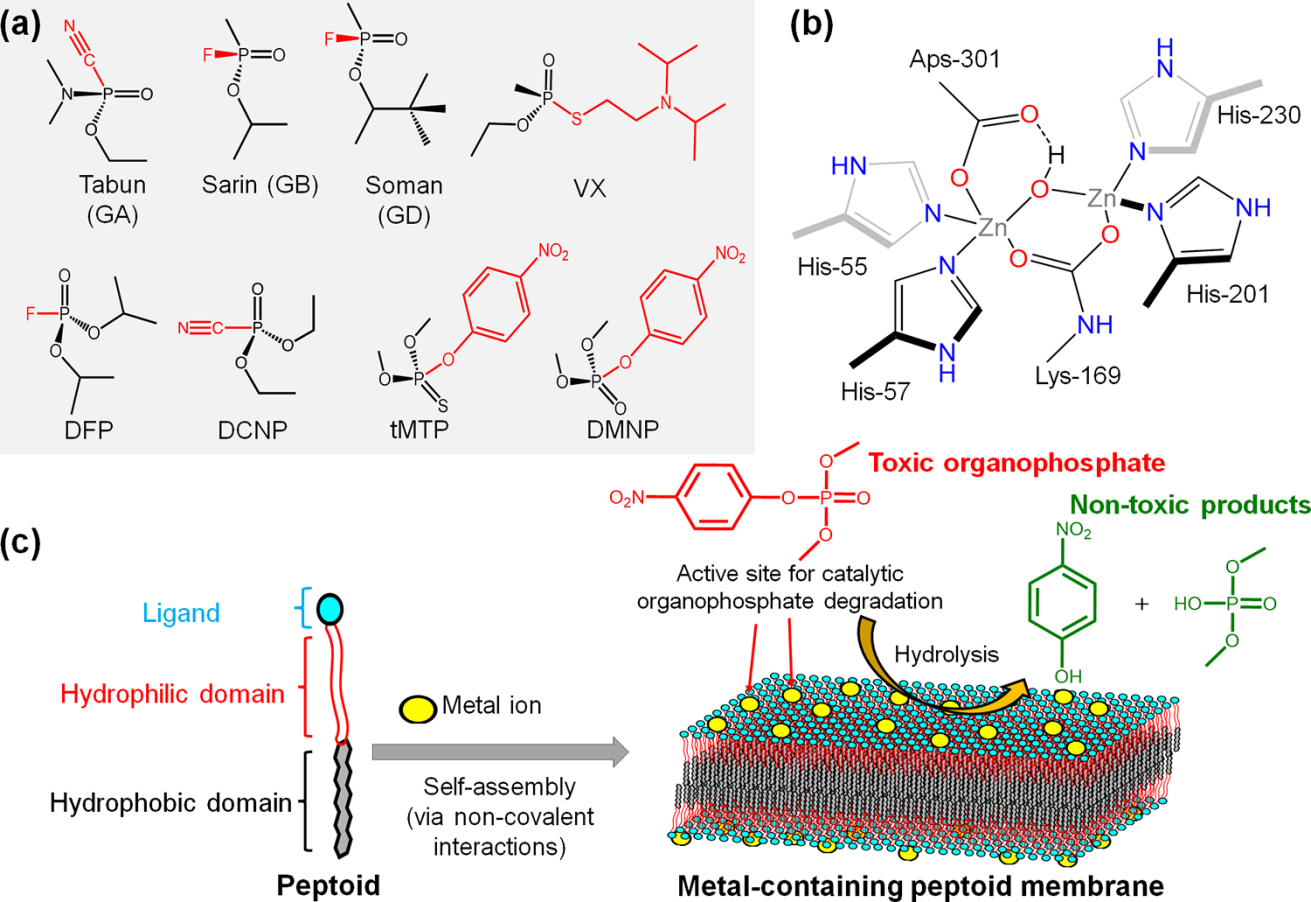
(a) Chemical structures for some common organophosphates (OPs) with leaving groups marked in red. (b) Active site model in phosphotriesterase. Adapted with permission from ref (9) Copyright 2020 American Chemical Society. (c) Assembly of sequence-defined peptoids into metal-containing membranes for effective degradation of DMNP.

Organophosphates (OPs) are a human-made family of chemicals that contain a phosphate ester and a series of highly reactive leaving groups (Figure 1a). They are recognized as one of the most neurotoxic chemicals and are widely utilized as pesticides in divergent industries and even as chemical warfare agents.^1−3^ Acute or chronic exposure to OP substances can result in severe health risks to humans and other living organisms. They can specifically and irreversibly inhibit the acetylcholinesterase (AChE) enzyme, leading to an accumulation of acetylcholine in the nervous system, thereby causing severe harm (respiratory, nervous, hepatic, etc.) and eventually death.^4^ Moreover, the accumulation of OPs in the environment can disrupt microbial communities, decreasing soil fertility.^2^ Due to their constant threat to society, developing appropriate chemical decontamination methods to mitigate nerve agents in an effective manner has been particularly crucial over the past decade. It is, therefore, vital to design biocompatible and robust materials that can efficiently degrade OPs into environmentally benign products.^5,6^

Numerous natural enzymes have been studied for the purpose of degrading OPs; they are able to catalyze the hydrolysis of a wide range of OPs and achieve a high catalytic turnover with orders of magnitude efficiency.^7^ One of the most efficient and well-studied enzymes explored is *Pseudomonas diminuta* phosphotriesterase (PTE), which shows excellent hydrolytic efficiency toward the insecticide paraoxon (*k*_cat_/*K*_M_ ≈ 10^8^ M^–1^ s^–1^).^8^ However, either in wild-type or recombinant strains, the enzyme is difficult to prepare and is readily denatured and inactivated upon exposure to environmental conditions.^1^ Accordingly, they are costly and, thus, not economical for deployment on a large scale.

To overcome the shortcomings mentioned above and retain high catalytic efficiency, researchers have devoted tremendous effort to develop PTE-mimetic catalysts. Many studies have focused on mimicry of the PTE active site, which contains two zinc ions ligated by an aspartic acid (Asp-301), four histidines (His-55, His-57, His-201, and His-230), a carboxylated lysine (Lys-169), and a hydroxide ion (Figure 1b).^7,9^ During hydrolysis, the binding between the phosphorus center of the OP and the active site metal ions and the intramolecular nucleophilic attack facilitated by neighboring amino acids are critical for the cleavage of phosphate ester bonds.^7,10^ For example, various biomimetic catalysts have been developed by mimicking the active site of PTEs using small-molecule metal-binding ligands that preclude the precipitation of metal hydroxide and simultaneously afford active hydroxo–metal complexes.^11,12^ Direct usage of the soluble monomeric metal complexes is effective^13,14^ but is difficult to use for many applications, since they do not enable facile separation from the products and reactants. This disadvantage, however, can be conquered by immobilizing the complexes into various supports, such as porous organic polymers^15,16^ or molecularly imprinted polymers.^16,17^ However, the embedment of functional sites in the interior area of these materials reduces their accessibility to the substrate, dramatically impacting the overall catalytic efficiency. Thus, it is still a standing challenge to produce materials with optimal active site placement that are effective for catalytic reactions.

Peptoid-based membrane-mimetic nanosheets (called two-dimensional (2D) nanomembranes) are a type of recently developed and highly programmable 2D nanomaterials.^18^ These free-standing nanomembranes have high surface areas and are highly stable, e.g., stable-to-extreme pH conditions: pH 1.0–11.0 and 60 °C overnight heating in aqueous and mixed solvent environments.^18^ Because peptoids are sequence-defined and easy to synthesize, we previously demonstrated that peptoid membranes are highly functionalizable, where attaching a broad range of functional groups at various locations in the peptoid sequence leaves the general membrane structure intact.^19^ A co-crystallization approach has also been reported to distribute functional groups within the nanomembranes at controlled densities.^18,19^ Moreover, the surface chemistry of these peptoid membranes can be programmed for the covalent attachment of specific functional groups, including molecular probes, to achieve long-range ordering for superior properties,^20,21^ such as high photostability, detection sensitivity, and enhanced catalytic performance. Therefore, these peptoid membranes offer a unique platform for precisely displaying the functional metal sites on the membrane surfaces.^22^

**Figure 2:**
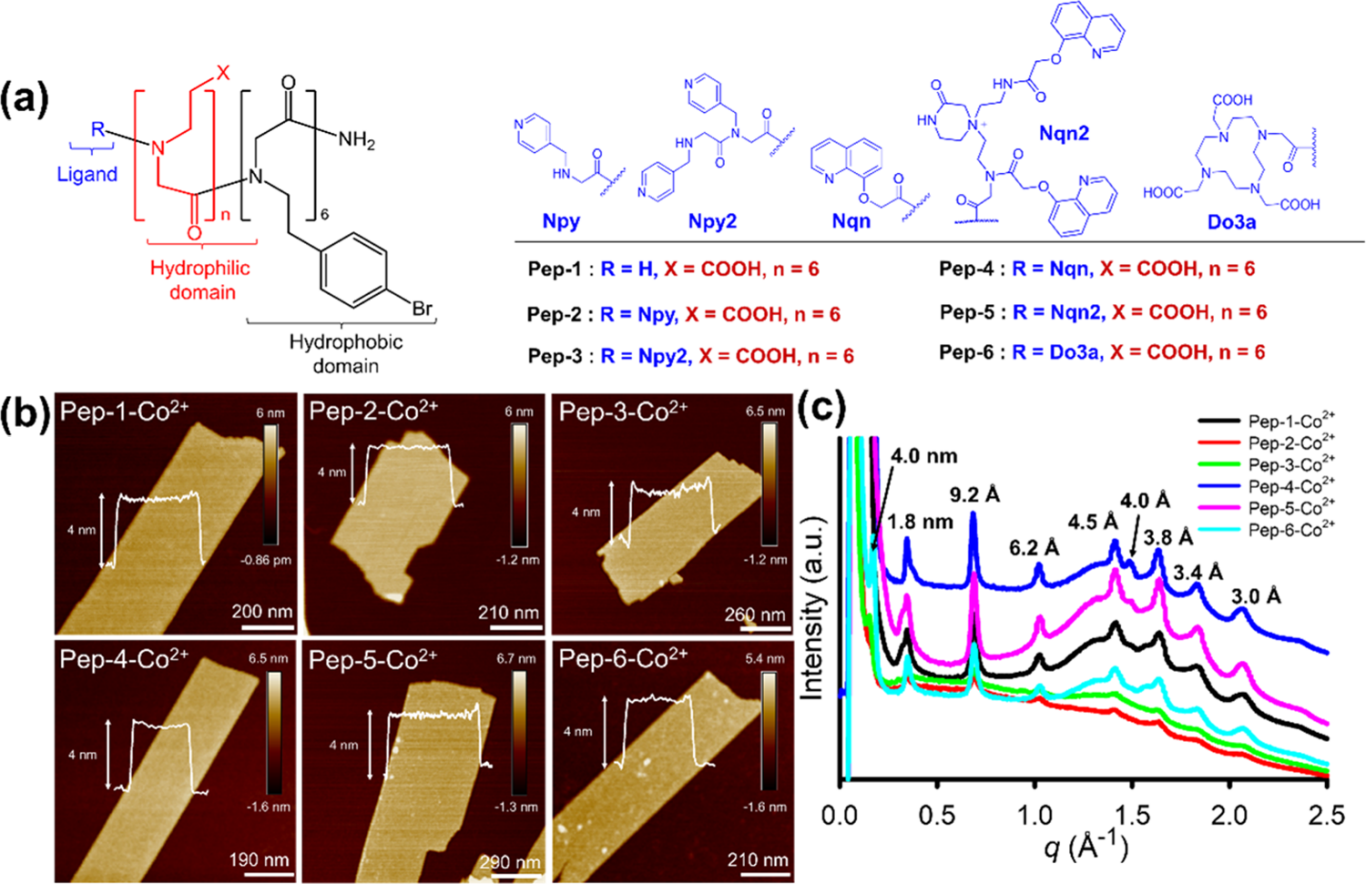
Characterization of Co^2+^-containing nanomembranes assembled from amphiphilic peptoids. (a) Schematic representation of peptoid molecules. (b) AFM topographic images displaying the assembled structures of the peptoids and Co(BF_4_)_2_. (c) XRD spectra of peptoid–Co^2+^ membranes. Characteristic peaks were analyzed based on the equation *d* = 2π/*q*.

Herein, to create a high-performance biomimetic catalyst using peptoid membranes, our design has focused on two major features: architectural control of the crystalline 2D membrane and the precise identity, location, and density of active domains.^23−25^ We aimed to functionalize the hydrophilic termini of membrane-forming peptoids with metal coordinating groups to coordinate to Lewis acidic metal ions and align them on the nanomembrane surface, thereby favoring substrate binding and diffusion (Figure 1c). Moreover, fine-tuning the chemical microenvironment close to the metal centers enables us to investigate their effects on the catalytic behavior without disruption of the assembled peptoid nanostructure, which is governed by crystalline hydrophobic interactions.^18^ We therefore synthesized a set of amphiphilic peptoids with different metal-coordination ligands (pyridyl (Npy), 8-hydroxyquinoline (Nqn), and a macrocyclic chelator (Do3a)) and hydrophilic linker units (Figure 2a). Inspired by previous studies with PTE, where its native metal substituted by a cobalt ion (Co^2+^) was found to be the most active form,^26,27^ we first selected Co^2+^ as our metal cation model and performed the peptoid assembly in the presence of Co^2+^ cations to generate Co^2+^-containing nanomembranes. The hydrolytic activity of peptoid assemblies was systematically investigated with a model substrate, dimethyl 4-nitrophenyl phosphonate (DMNP), which is a well-known nerve agent simulant.^28^ Our results showed that certain Co^2+^-containing peptoid membranes are highly efficient for catalytic degradation of DMNP, and we demonstrated precise control over the density and local environment of Co^2+^ active sites for tuning their catalytic activities. These findings highlight that peptoid membranes offer a highly robust and programmable platform for developing highly efficient biomimetic catalysts that could potentially be used for degrading OP compounds and other catalytic reactions.

## Results and Discussion

### Synthesis and Characterization of Self-Assembled Peptoid Membranes

**Figure 3:**
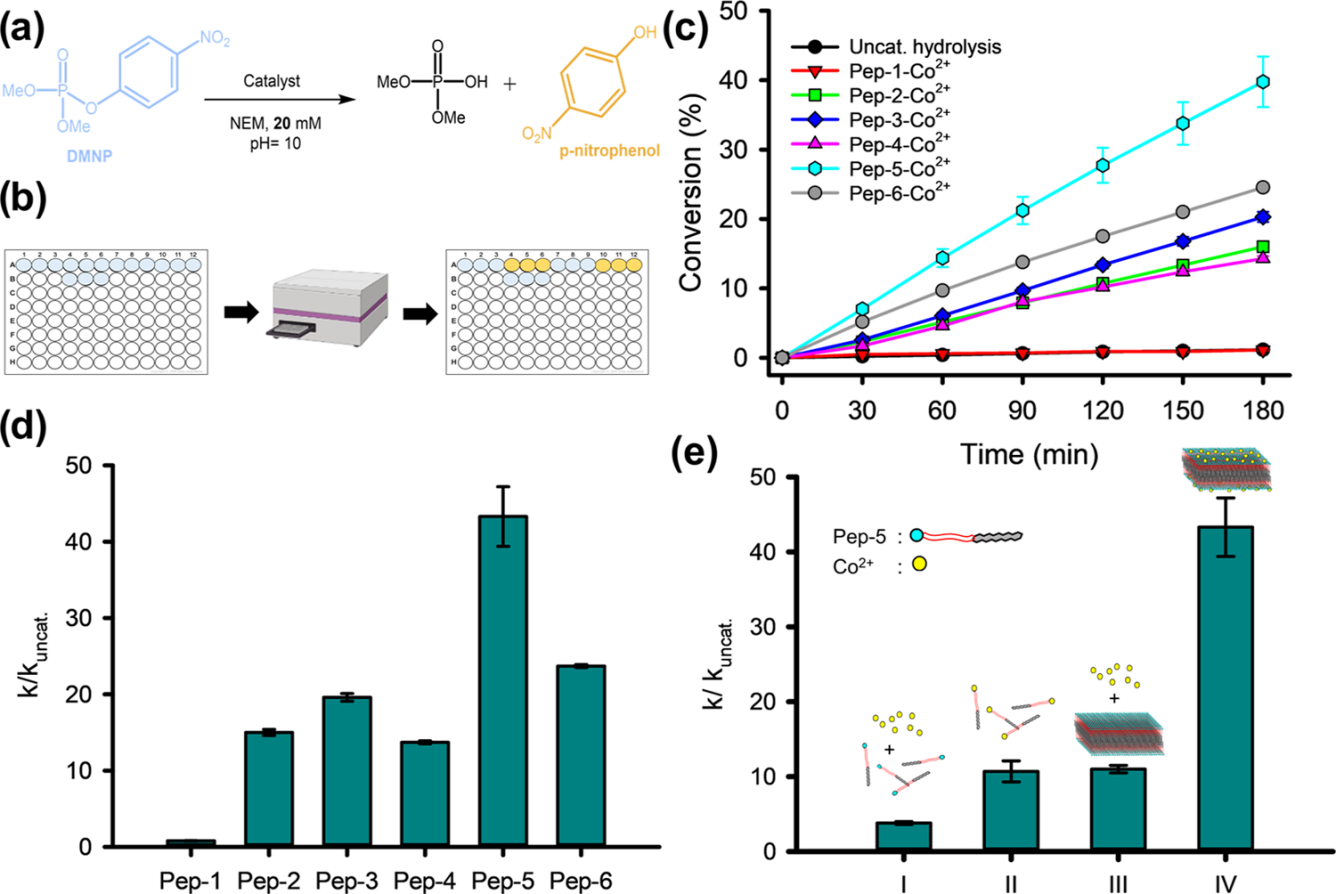
DMNP hydrolysis induced by various Co^2+^-containing peptoid membranes. (a) Schematic representation of DMNP hydrolysis in NEM buffer. (b) Representation of a high-throughput setup for testing the activity of peptoid membranes toward DMNP hydrolysis. (c) Hydrolysis profile of DMNP hydrolysis catalyzed by Co^2+^-containing membranes assembled from various peptoids (**Pep-1**–**Pep-6**). (d) Effects of the number of ligands and their chemistries on the activity of membrane-triggered DNMP hydrolysis. (e) Effect of membrane crystallinity on the membrane-triggered DMNP degradation (I: amorphous **Pep-5**–Co^2+^ mixture, II: amorphous **Pep-5**–Co^2+^ complex, III: **Pep-5** membrane–Co^2+^, IV: **Pep-5**–Co^2+^ membrane. Hydrolysis conditions for c–e: [DMNP] = 0.25 mM, [peptoid membrane] = 0.125 mM, [NEM] = 20 mM, pH 10).

**Figure 4:**
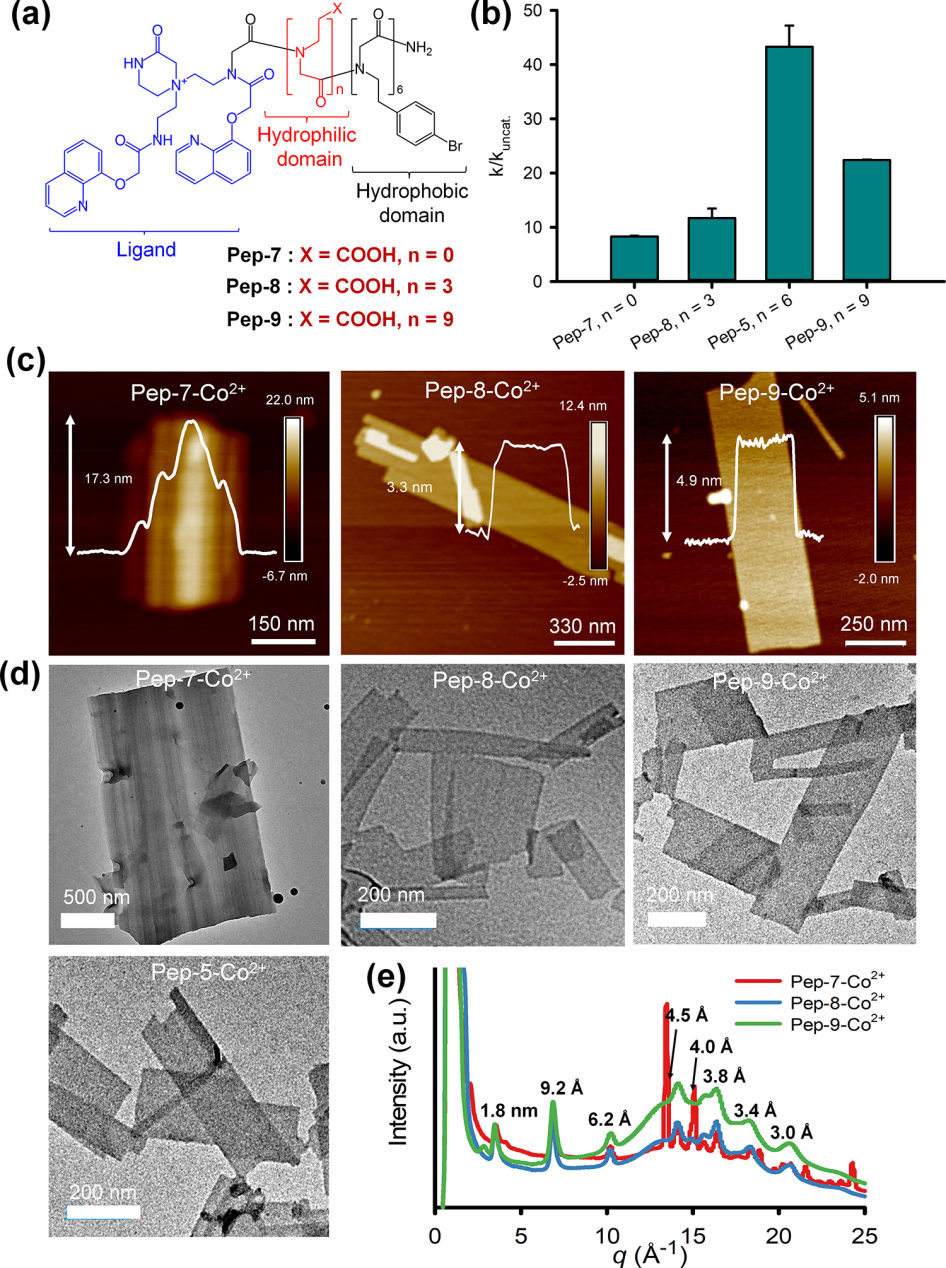
Effect of the hydrophilic lengths of peptoid chains on the catalytic performance of nanomembranes in DMNP degradation. (a) Chemical structures of peptoids with various lengths of the hydrophilic domain. (b) Influence of backbone lengths on the rate enhancement of membrane-catalyzed DMNP degradation. (c) AFM images of Co^2+^-containing nanomembranes assembled from **Pep-7**, **Pep-8**, and **Pep-9**. The insets are height profiles showing the height of formed peptoid–Co^2+^ membranes. (d) TEM images of Co^2+^-containing nanomembranes assembled from **Pep-5**, **Pep-7**, **Pep-8**, and **Pep-9**. (e) XRD results of **Pep-7**–Co^2+^, **Pep-8**–Co^2+^, and **Pep-9**–Co^2+^ membranes. Characteristic peaks were analyzed based on the equation *d* = 2π/*q*.

In order to produce metal-containing nanomembranes in which metal complexes that mimic enzyme active sites are precisely placed on the membrane surfaces suitable for DMNP hydrolysis, we designed a number of membrane-forming peptoids containing various ligands. We focused on varying both the metal-binding ligand itself and the adjacent hydrophilic linker domain while maintaining the same hexapeptoid hydrophobic domain for the preparation of peptoid membrane catalysts. Ligand-containing peptoids were synthesized using the submonomer solid-phase synthesis method reported previously.^22^ These peptoids contain six Nbrpe = *N*-([2-(4-bromophenyl)ethyl]glycine) in the hydrophobic domain and a varied number of Nce = *N*-(2-carboxyethyl)glycine in the hydrophilic domain. The presence of six Nbrpe units has been demonstrated to be favorable for nanomembrane formation to form a crystalline hydrophobic core based on enhanced aromatic stacking interactions.^29−32^ Three different ligands, based on Npy, Nqn, and Do3a units,^33−36^ were used to create metal-binding sites (Figure 2a), generating peptoids **Pep-1**–**Pep-6**. Many studies (i.e., Maayan et al.^37−39^ and Chen et al.^22^) previously demonstrated that the backbone length and a noncoordinating side chain located adjacent to an active site within the peptoid chain could have significant impacts on the catalytic reactions. Thus, we further designed and synthesized **Pep-7** to **Pep-15** with varying backbone lengths and side-chain chemistries (Figures 3a and 4a). Detailed synthesis and characterization of these peptoids, including their ultra-performance liquid chromatography-mass spectrometry (UPLC-MS) data, are shown in the Supporting Information (Figures S1–S15). Notably, during the synthesis of **Pep-5** and its derivatives (**Pep-7**–**Pep-15**), we encountered an unanticipated side reaction involving the formation of an unusual cyclic product in very high yield. Particularly, in the acylation step, the intermediate tris-bromoactylated peptoid underwent an intramolecular cyclization reaction with the N-terminal tertiary amine in the linker domain, resulting in a quaternary salt of a cyclic monoketopiperazine, as illustrated in Figure S16.^40^ It is likely that this reaction results in a mixture of two regioisomers (Figure S17), but for the sake of clarity and ease of understanding, we have presented only one possible structure in the main text.

**Figure 5:**
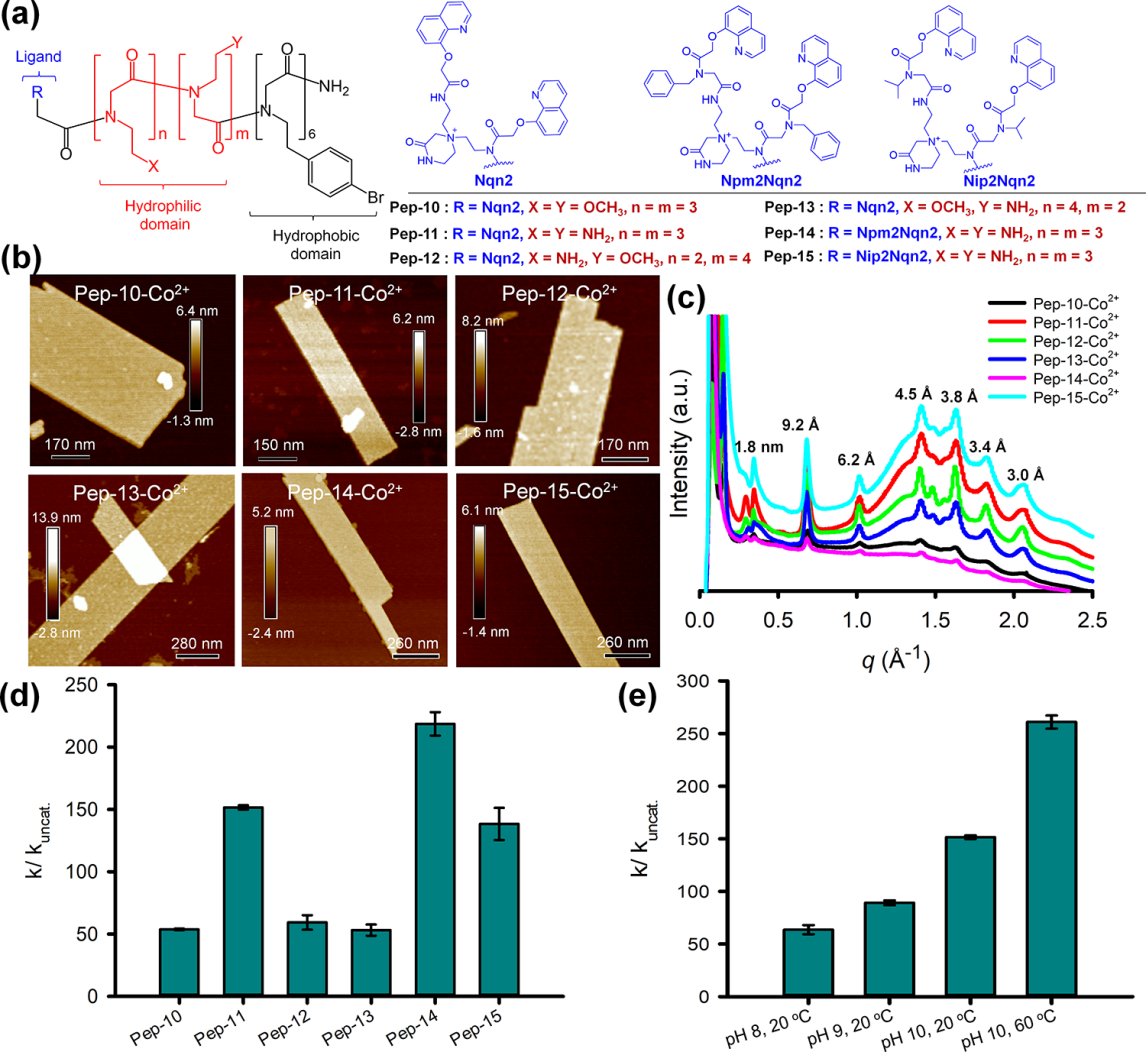
Membrane-catalyzed DMNP degradation under various conditions. (a) Structure representative of peptoid sequences. (b) AFM images of Co^2+^-containing membrane assembled from **Pep-10** to **Pep-15**. (c) XRD results of the Co^2+^-containing membrane assembled from **Pep-10** to **Pep-15** exhibit similar peaks, suggesting that they all possess similar framework structures. (d) Effect of the hydrophilic domain and microenvironment of active sites on the rate enhancement of DMNP hydrolysis. (e) Effect of pH and temperature on the rate enhancement of DMNP hydrolysis triggered by **Pep-11**–Co^2+^ membranes.

**Figure 6:**
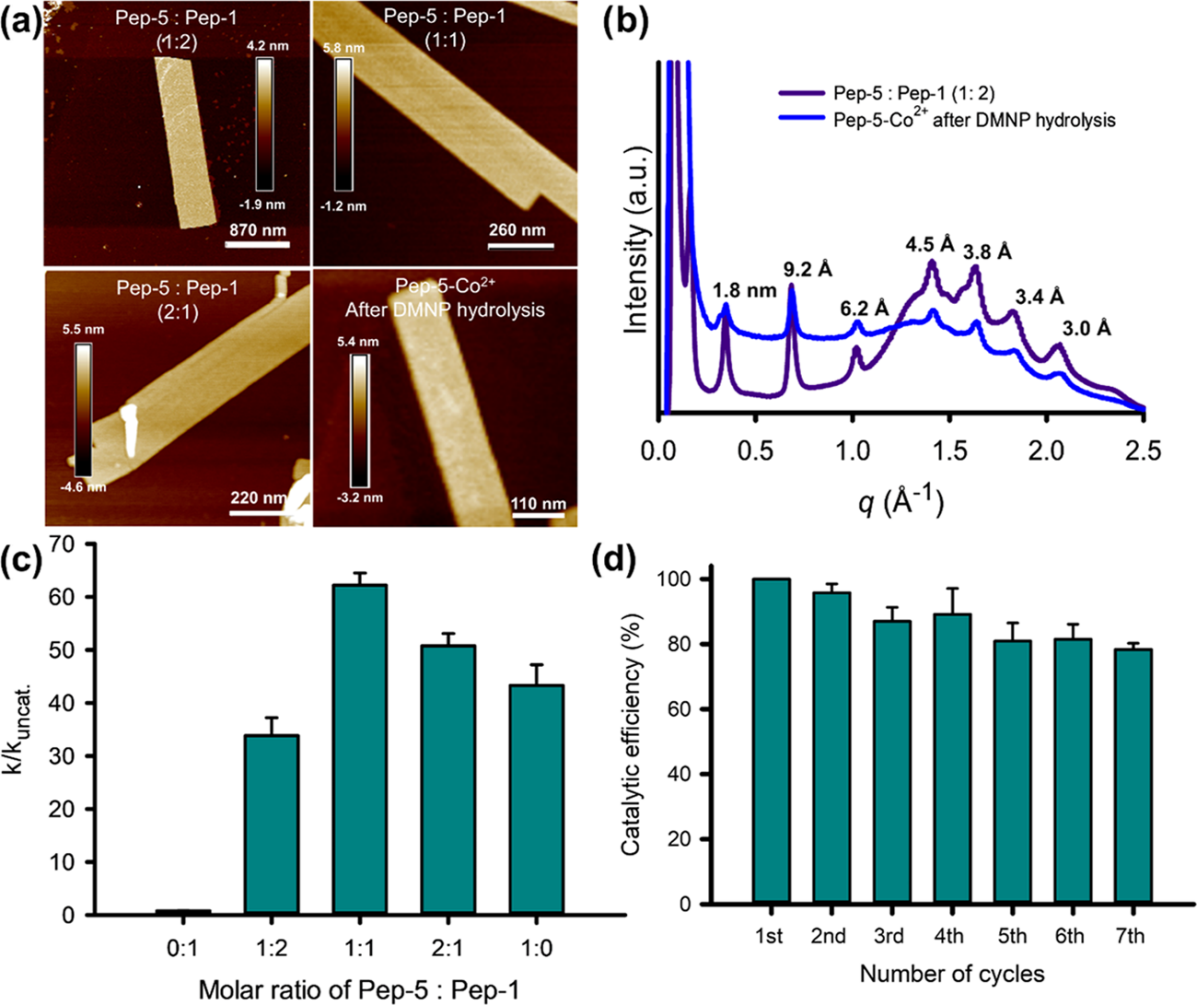
Influence of the active site density of peptoid–Co^2+^ membranes on DMNP hydrolysis and their reusability. (a) AFM images of membranes assembled from various mixtures of **Pep-5** and **Pep-1**, as well as the **Pep-5**–Co^2+^ membrane after the 3^rd^ catalytic cycle. (b) XRD spectra of the Co^2+^-containing membrane resulting from the self-assembly of **Pep-5** and **Pep-1** (**Pep-5/Pep-1** = 1:2 equiv), and the **Pep-5**–Co^2+^ membrane after the 3^rd^ hydrolytic cycle. All of the observed XRD peaks closely match the typical peaks of crystalline peptoid membranes, indicating that our peptoid membranes possess high crystallinity and stability. (c) Effect of Nqn–Co^2+^ active site density controlled by varying the **Pep-5**-to-**Pep-1** molar ratio on the membrane-catalyzed DMNP degradation. (d) Catalytic efficiency of **Pep-5**–Co^2+^ membranes with seven subsequent cycles.

To obtain Co^2+^-containing nanomembranes, peptoids were mixed with Co(BF_4_)_2_ in water/acetonitrile = 1:1 (v/v), followed by slow evaporation at 4 °C to induce the crystallization of the Nbrpe hydrophobic block. Atomic force microscopy (AFM) images and transmission electron microscopy (TEM; Figures 2b, 4c, 5b, 6a, and S18) clearly illustrated that all peptoids assembled into discrete 2D nanomembranes. Membrane thickness was determined around 4 nm, which agrees with our previous results for the defined nanomembrane under dry conditions.^18^

To further investigate the self-assembled membrane structures, we carried out X-ray diffraction (XRD) characterization that demonstrated the generation of highly crystalline materials with similar XRD patterns to previously reported peptoid-based nanomembranes (Figures 2c, 4e, 5c, and 6b).^18^ The peak showing a spacing of 4.5 Å is associated with the ordered alignment of peptoid backbone chains. The 1.8 nm spacing is the distance between two peptoid backbones packed inside membranes with Nbrpe facing each other. The spacing of 3.0 Å corresponds to the distance between two adjacent side-chain residues along the backbone chain direction (i.e., N···N distance) of a *cis*-conformation peptoid. The significant peaks at 4.0, 3.8, and 3.4 Å suggest the presence of extensive π-stacking interactions among Nbrpe groups within peptoid membranes.^18,32^ These XRD results show that functionalizing the N-terminus with a wide variety of ligands with bound Co^2+^ ions readily yields functionalized membranes with similar framework structures, further confirming the high tunability of these 2D nanomaterials for biomimetic catalysis.

To validate the coordination between Co^2+^ and peptoids within nanomembranes, we further performed ultraviolet–visible (UV–vis) and Fourier transform infrared (FTIR) spectroscopy studies. First, we conducted an investigation into the ability of Co^2+^ to form a complex with free peptoids in the solution. Upon addition of Co(BF_4_)_2_ to peptoid solutions in acetonitrile, the absorbance at 260 nm for **Pep-2** and **Pep-3**, 315 nm for **Pep-4** and **Pep-5**, and 271 nm for **Pep-6** showed an increase, indicating the formation of peptoid–Co^2+^ complexes (Figure S19). These titration results were used to construct metal-to-peptoid ratio plots, which revealed the binding stoichiometry of peptoid: Co^2+^ as 1:0.25 (for **Pep-2** and **Pep-4**), 1:0.5 (for **Pep-3** and **Pep-5**), and 1:1 (for **Pep-6**).

Then, we investigated the binding of Co^2+^ with peptoids to form membranes. The membranes were prepared by coassembling Co^2+^ with the corresponding peptoids, following the binding ratios mentioned earlier. The amount of metal ions bound to these nanomembranes was determined using Eriochrome Black T as a complexometric indicator.^41,42^ The results demonstrated that 65 to 95% of the initially added metal was bound to the assembled membranes (Figures S20–21 ). Besides, the appearance of the characteristic bands at 533 and 423 cm^–1^ in the FTIR spectrum of the **Pep-5**–Co^2+^ membrane assigned to υ (Co–N) provides further evidence for the chelation of the peptoid and metal (Figure S22).^43^ The presence of Co^2+^ within peptoid membranes was further confirmed by the X-ray photoelectron spectroscopy (XPS) (Figure S23) and TEM/energy dispersive spectroscopy (EDS, Figure S24) analyses of **Pep-5**–Co^2+^ membranes.

To confirm that these Co^2+^-containing nanomembranes are stable and suitable for catalytic degradation of DMNP, we tested their stability under a variety of conditions, including exposure to elevated temperatures and organic solvents. As displayed in Figure S25a,d, AFM images showed that peptoid membranes remained intact after heating their aqueous solution to 60 and even 90 °C for over 2 h. They also showed good stability in ethanol for 2 h and in *N*-ethylmorphine (20 mM, pH 10) for over a week. Furthermore, **Pep-5**–Co^2+^ membranes recovered from both thermal and organic solvent treatments showed minimal changes in their catalytic degradation of DMNP (Figure S25e), confirming that these metal-containing membranes are highly robust. The high stability results allow us to investigate the catalytic activity of these nanomembranes in a range of environmental conditions as well as reuse them for the degradation of DMNP.

### Catalytic Activity of Peptoid–Metal Nanomembranes

We examined the catalytic efficiency of the functionalized peptoid nanomembranes in an aqueous solution against DMNP by UV–vis to monitor the release of *p*-nitrophenolate (λ_abs_ = 400 nm), as shown in Figures 3a and S26a. Nanomembrane solutions were assayed at a peptoid concentration of 0.125 mM and a DMNP concentration of 0.25 mM. To enable rapid screening, all reactions were conducted in 96-well plates and read in a commercial microplate reader (Figure 3b).^44^ This method requires less than 200 μg of the material and can examine many different catalysts and conditions in a rapid time frame and in a high-efficiency manner.

#### Effect of Ligand Chemistry

Because the coordination of ligands with metal centers is a key feature of metalloenzyme active sites, such as PTEs,^45−47^ herein, we used different ligands to coordinate with metal cations to mimic active sites of natural enzymes. As shown in Figure 2a, six different peptoid sequences (**Pep-1**–**Pep-6**) with different numbers and chemistries of ligands were synthesized. Npy, Nqn, and Do3a were selected as ligands because they are known to coordinate transitional metal cations.^22,48,49^ The Co^2+^ cation was selected because of a notable enhancement in the activity of PTE when native Zn^2+^ was substituted by Co^2+^. The catalytic activities of Co^2+^-containing peptoid nanomembranes made from **Pep-1** to **Pep-6** were tested in NEM buffer (pH 10) at room temperature. These are common conditions commonly utilized to evaluate enzyme mimics for DMNP degradation.^28,50^Figure 3c illustrates the conversion profile of DMNP catalyzed by the Co^2+^-containing membranes. Direct analysis reveals variable first-order rate constants, turnover number (TON), and turnover frequency (TOF), as described in Table 1, Figure S27, and Table S1. Negligible catalytic performance was found with **Pep-1**–Co^2+^, similar to the uncatalyzed background hydrolysis reaction (Figure 3d). In contrast, significant catalytic enhancements relative to the background (*k*_cat_ = 65.3 × 10^–6^ min^–1^, Table 1, entry 1) were observed with all of the other peptoids. The observed activity acceleration followed the order **Pep-4**–Co^2+^ (13.7-fold) ≤ **Pep-2**–Co^2+^ (15.0-fold) < **Pep-3**–Co^2+^ (19.6-fold) < **Pep-6**–Co^2+^ (23.7-fold) < **Pep-5**–Co^2+^ (43.3-fold) (Table 1, entries 2–7). These results indicate that the introduction of an optimal ligand is crucial for rate enhancement.^51,52^ The most efficient peptoid membrane catalyst in this set is **Pep-5**–Co^2+^membranes where the ligand of **Pep-5** consists of two Nqn groups.

**Table 1:** Summary of DMNP Hydrolysis Activities Triggered in NEM Buffer under Various Conditions ([DMNP] = 0.25 mM and [NEM] = 20 mM, pH 10)

entries	catalyst	pH	*k*_cat_ (×10^–6^ min^–1^)	*k*_cat_/*k*_uncat_
1	none	10	65.3 ± 1.7	1
2	**Pep-1**–Co^2+^ membrane	10	54.2 ± 2.8	0.8 ± 0.04
3	**Pep-2**–Co^2+^ membrane	10	980 ± 27	15.0 ± 0.4
4	**Pep-3**–Co^2+^ membrane	10	1280 ± 33	19.6 ± 0.5
5	**Pep-4**–Co^2+^ membrane	10	897 ± 17	13.7 ± 0.2
6	**Pep-5**–Co^2+^ membrane	10	2830 ± 250	43.3 ± 3.9
7	**Pep-6**–Co^2+^ membrane	10	1550 ± 13	23.7 ± 0.2
8	amorphous **Pep-5**–Co^2+^	10	247 ± 17	3.8 ± 0.2
9^a^	amorphous **Pep-5**–Co^2+^ complex	10	701 ± 89	10.7 ± 1.4
10^b^	soluble **NpmNqn2**–Co^2+^ complex	10	615 ± 25	9.4 ± 0.4
11^c^	**Pep-5** membrane–Co^2+^	10	720 ± 33	11.0 ± 0.5
12	**Pep-5**–Cu^2+^ membrane	10	3200 ± 150	49.0 ± 2.2
13	**Pep-5**–Zn^2+^ membrane	10	2140 ± 56	32.7 ± 0.8
14	**Pep-7**–Co^2+^ membrane	10	542 ± 8	8.3 ± 0.1
15	**Pep-8**–Co^2+^ membrane	10	764 ± 11	11.7 ± 0.1
16	**Pep-9**–Co^2+^ membrane	10	1470 ± 7	22.4 ± 0.1
17	**Pep-10**–Co^2+^ membrane	10	3510 ± 38	53.7 ± 0.6
18	**Pep-11**–Co^2+^ membrane	10	9900 ± 830	152 ± 1.7
19	**Pep-12**–Co^2+^ membrane	10	3880 ± 380	59.3 ± 5.8
20	**Pep-13**–Co^2+^ membrane	10	3470 ± 290	53.1 ± 4.5
21	**Pep-14**–Co^2+^ membrane	10	14300 ± 620	219 ± 9.4
22	**Pep-15**–Co^2+^ membrane	10	9040 ± 840	139 ± 12.9
23	**Pep-11**–Co^2+^ membrane	9	5820 ± 150	89.1 ± 2.3
24	**Pep-11**–Co^2+^ membrane	8	4150 ± 280	63.6 ± 4.3
25	**Pep-11**–Co^2+^ membrane, 60 °C	10	17050 ± 406	261 ± 6.2
26	**Pep-5**-**Pep-1**–Co^2+^ (1:2) membrane	10	2210 ± 313	33.8 ± 4.8
27	**Pep-5**-**Pep-1**–Co^2+^ (1:1) membrane	10	4070 ± 149	62.2 ± 2.3
28	**Pep-5**-**Pep-1**–Co^2+^ (2:1) membrane	10	3320 ± 150	50.8 ± 2.3
29^d^	**Pep-5**–Co^2+^ membrane, 2nd cycle	10	2700 ± 77	41.5 ± 1.2
30^d^	**Pep-5**–Co^2+^ membrane, 3rd cycle	10	2620 ± 122	40.3 ± 1.9
31^d^	**Pep-5**–Co^2+^ membrane, 4th cycle	10	2610 ± 224	40.2 ± 3.4
32^d^	**Pep-5**–Co^2+^ membrane, 5th cycle	10	2550 ± 157	39.2 ± 2.4
33^d^	**Pep-5**–Co^2+^ membrane, 6th cycle	10	2420 ± 132	37.2 ± 2.0
34^d^	**Pep-5**–Co^2+^ membrane, 7th cycle	10	2400 ± 54	36.9 ± 0.8

a A mixture of amorphous **Pep-5** and Co(BF_4_)_2_ premixed for a week.

b A mixture of soluble NpmNqn2 and Co(BF_4_)_2_ premixed for a week.

c A mixture of **Pep-5** membrane and Co(BF_4_)_2_ premixed for a week.

d Co^2+^-containing membranes were recovered following the specific protocol reported in the Supporting Information.

#### Effect of Peptoid–Co^2+^ Membrane Crystallinity

To highlight the importance of the high crystallinity of **Pep-5**–Co^2+^ membranes in DMNP degradation, we conducted DMNP degradation using amorphous aggregates of **Pep-5**–Co^2+^ complexes (Figure 3e). The amorphous aggregates were prepared by rapidly mixing **Pep-5** with Co(BF_4_)_2_ in acetonitrile solvent, followed by lyophilization and resuspension in a NEM buffer. When this material was used for DMNP hydrolysis, it displayed a hydrolytic activity that was 11.4-fold lower than that of crystalline **Pep-5**–Co^2+^ membranes (Table 1, entry 8). To ensure the binding of ligands with Co^2+^ cations and further highlight the importance of membrane crystallinity in DMNP degradation, we also prepared another **Pep-5**–Co^2+^ complex sample by incubating **Pep-5** with Co(BF_4_)_2_ for a week to ensure the coordination of Nqn ligands with Co^2+^. Likewise, this second sample displayed considerably reduced hydrolytic activity in contrast to the crystalline **Pep-5**–Co^2+^ membranes (Table 1, entry 9), yet it closely resembled the activity of the soluble **NpmNqn2**–Co^2+^ complex, which mimics the active site of **Pep-5**–Co^2+^ (Table 1, entry 10, and Figure S28). These findings suggest the ordering of ligand–Co^2+^ active sites as a result of membrane crystallinity, which is critical for achieving a high efficiency in DMNP degradation. Such a phenomenon is consistent with our recent study in developing crystalline peptoid assemblies as biomimetic catalysts in lignin degradation.^22^ To highlight the importance of the membrane catalyst preparation process, we also incubated Co^2+^-free **Pep-5** membranes with an aqueous solution of Co(BF_4_)_2_ in a **Pep-5**–Co^2+^ molar ratio of 1:0.5 for a week. This obtained **Pep-5** membrane–Co^2+^ complex exhibited a *k*_cat_/*k*_uncat_ of 11 (Table 1, entry 11), which is 3.9-fold lower than that of crystalline **Pep-5**–Co^2+^ membranes. We reason that the close packing of Nqn ligands within Co^2+^-free **Pep-5** membranes limits their efficiency of coordinating with Co^2+^, thus reducing the number of ligand–Co^2+^ active sites as well as their ordering within membranes for catalytic DMNP degradation. In conclusion, the coassembly of peptoids and Co^2+^ cations to form crystalline **Pep-5**–Co^2+^ membranes was found to be the best way to produce highly active membrane catalysts.

#### Effect of Metal Cations

During the catalysis of natural enzymes, metal cations such as Zn^2+^ act as Lewis acids to catalyze hydrolysis reactions; thus, the choice of metal cations is expected to play an important role in the DNMP degradation catalyzed by metal-containing peptoid membranes. Accordingly, we investigated how other transition metal ions, such as Cu^2+^ and Zn^2+^, influence the performance of membranes in DMNP degradation. For that, we synthesized metal-containing **Pep-5** membranes in the presence of Cu^2+^ or Zn^2+^ using a similar assembly approach (see the Supporting Information for details) and studied their hydrolytic activity against DMNP. In kinetic profiles depicted in Figure S25c, crystalline **Pep-5**–Cu^2+^ membranes exhibited an increased activity (*k*_cat_/*k*_uncat_ = 49.0) compared to crystalline **Pep-5**–Co^2+^ membranes (*k*_cat_/*k*_uncat_ of 43.3), while **Pep-5**–Zn^2+^ membranes displayed a slight reduction in hydrolytic activity (*k*_cat_/*k*_uncat_ of 32.7; Table 1, entries 12 and 13) reactivity. Both Cu^2+^ and Zn^2+^ bound to **Pep-5** with a 0.5 molar ratio, and the amounts of metals loaded were 95 and 96% for **Pep-5**–Cu^2+^ and **Pep-5**–Zn^2+^, respectively, which are comparable to that of **Pep-5**–Co^2+^ (Figure S29). Differences in the rate enhancement could be due to the electronegativity of the metal cations, which also increases following the order Zn^2+^ (χ = 1.65) < Co^2+^ (χ = 1.88) < Cu^2+^ (χ = 1.90).^26^ This result is consistent with previously reported enzyme mimetic studies in which both experimental and simulated results showed that the selection of metal cations with a higher electronegativity led to a better catalytic activity.^26,53,54^ Our findings suggest that the catalytic activity of metal-containing peptoid membranes can be further optimized by using metal cations possessing a higher electronegativity.

#### Effect of Hydrophilic Linker Length

Next, to explore the impact of the hydrophilic linker length of peptoids on membrane hydrolytic activity, a series of **Pep-5** derivative peptoids containing different numbers (*n*) of Nce groups (Figure 4a) were synthesized and used for the assembly of crystalline membranes in the presence of Co^2+^ cations (see the Experimental Section for details). As depicted in Figure 4b, all Co^2+^-containing peptoid membranes are effective in DMNP degradation. Among them, membranes assembled from **Pep-5** with *n* = 6 Nce groups displayed the highest efficiency. Conversely, membranes assembled from sequences with fewer Nce groups (**Pep-7**, *n* = 0; **Pep-8**, *n* = 3) or more Nce groups (**Pep-9**, *n* = 9) exhibited lower efficiency in DMNP degradation (Table 1, entries 14–16). These findings suggest that maintaining an optimal distance between Nqn–Co^2+^ active sites and the hydrophobic cores of peptoid membranes is crucial for achieving a high catalytic efficiency.

The morphologies of these nanomembranes were confirmed by AFM and TEM characterization. Both AFM and TEM results showed that while both **Pep-8** and **Pep-9** formed nice nanomembranes as **Pep-5** did in the presence of Co^2+^ cations (Figure 4c,d) and XRD data of these peptoid–Co^2+^ membranes showed similar patterns (Figure 4e), **Pep-7** with *n* = 0 formed multilayer membrane structures and an obvious difference in some XRD peaks. Fluorescence images of these peptoid–Co^2+^ membranes further confirmed the dramatic feature of the **Pep-7**–Co^2+^ membranes by showing the largest aggregation among the **Pep-5**–Co^2+^, **Pep-7**–Co^2+^, and **Pep-8**–Co^2+^ membranes (Figure S30). Because the metal centers are positioned on the membrane surface, we reasoned that this multilayer stacking of membranes likely limited the exposure of Nqn–Co^2+^ active sites of peptoid membranes, thereby reducing its catalytic efficiency. These results indicate that the presence of the appropriate linker promotes the formation of free-standing nanomembranes with the maximum exposure of ligand–metal actives for catalysis.

#### Effect of the Active Site Microenvironment

In natural enzymes, the microenvironment surrounding their metal–ligand active sites plays a key role in regulating the catalytic reactions.^55,56^ For example, as shown in Figure 1b, in PTEs, the ligated aspartate, histidine, and lysine residues adjacent to the binuclear Zn^2+^ active site are critical for the catalytic OP hydrolysis process, suggesting the importance of amino groups around the active sites of **Pep-5**–Co^2+^ membranes for further improving their OP hydrolysis activity. To modulate the microenvironment of Nqn–Co^2+^ active sites, we synthesized **Pep-10** by having all Nce groups replaced with Nome (Nome = *N*-(2-methoxyethyl)glycine) and **Pep-11** with all Nae (Nae = *N*-(2-aminoethyl)glycine) groups in the polar domain (Figure 5a). AFM and TEM results confirmed the formation of nanomembranes (Figures 5b and S31). XRD results showed these membranes are highly crystalline and have peaks almost identical to those of other Co^2+^-containing peptoid membranes, suggesting a similar framework structure (Figure 5c). DMNP hydrolysis results showed that **Pep-10**–Co^2+^ exhibited an acceleration of *k*_cat_/*k*_uncat_ = 53.7, which is slightly higher than that of **Pep-5**–Co^2+^ membranes (*k*_cat_/*k*_uncat_ = 43.3), while **Pep-11**–Co^2+^ membranes showed a 152-fold acceleration (*k*_cat_/*k*_uncat_ = 152) (Figure 5d and Table 1, entries 17 and 18). These results confirmed the important role of amino groups in the design of peptoid membranes effective for catalytic degradation of OPs.

To test if the number and position of Nae groups are important for the catalytic DMNP degradation, we further synthesized **Pep-12** and **Pep-13** (Figure 5a), which both contain four Nome and two Nae groups but in different positions. As expected, in the presence of Co^2+^, both peptoids formed crystalline peptoid–Co^2+^ membranes whose structures are similar to those of **Pep-11**–Co^2+^ membranes. DMNP degradation results showed that the reduced numbers of Nae groups led to a decreased efficiency of peptoid–Co^2+^ membranes for DMNP degradation, suggesting that the number of amino groups in the membrane catalysts is more important than their locations in affecting the OP hydrolytic activity (Figure 5d and Table 1, entries 19 and 20).

It is well-known that substrate binding, governed in large part by the local hydrophobic environment, contributes significantly to the catalytic efficiency of enzymes and their mimics, such as peptides, polymers, and metal complexes.^7,57,58^ To mimic such a local hydrophobic environment to facilitate the binding with OPs, we designed and synthesized **Pep-14** (Figure 5a) by including aromatic Npm [Npm = *N*-(1-phenylmethyl)glycine] adjacent to the Nqn ligand. As expected, the assembly of **Pep-14** in the presence of Co^2+^ led to the formation of similar and highly crystalline Co^2+^-containing membranes confirmed by AFM and XRD results (Figure 5b,c). Interestingly, **Pep-14**–Co^2+^ membranes showed a nearly 219-fold acceleration in DMNP degradation (*k*_cat_/*k*_uncat_ = 219; Table 1, entry 21), suggesting the important role of the Npm-created microenvironment in promoting the catalytic efficiency. A possible reason for such enhancement could be due to the increased interaction of DMNP with Npm groups through π–π interaction.^59,60^ To test that, we further synthesized **Pep-15** by replacing Npm with a Nip [Nip = *N*-(isopropyl)glycine] group. While **Pep-15** formed a similar Co^2+^-containing membrane as **Pep-14** did, this membrane showed an almost identical hydrolysis activity to **Pep-11**–Co^2+^ membranes (Figure 5d and Table 1, entry 22), highlighting the important role of the local aromatic hydrophobic environment in the development of peptoid membrane catalysts for efficient OP degradation.

#### Effect of pH and Temperature

While natural enzymes are only active in the narrow physiological pH ranges, our Co^2+^-containing peptoid membranes are highly robust (Figure S24) and can be used under a wide range of conditions, such as different solution pH and elevated temperature, for catalytic performance. Because solution pH and temperature are known for their significant influence in the OP hydrolysis triggered by PTEs and biomimetic catalysts,^61^ herein, we further performed the **Pep-11**–Co^2+^ membrane-based DMNP hydrolysis in NEM buffer at three different solution pH (i.e., pH 8, pH 9, and pH 10) and also at an elevated temperature of 60 °C. Hydrolysis results showed that the increase of solution pH led to an increase in the hydrolysis rate following the order pH 8 (63.6-fold) < pH 9 (89.1-fold) < pH 10 (152-fold; Figure 5e and Table 1, entries 18, 23, and 24). This is consistent with previously reported OP hydrolysis triggered by Zn^2+^, Cu^2+^, Ni^2+^, and Co^2+^ complexes.^14,62,63^ As mentioned in these studies, the rate-determining step involves the nucleophilic attack of a hydroxide ion on the phosphorus center after the substrate binds with the metal ion. Unlike previous Zn^2+^ or Ag^+^ complexes owning hydroxyl groups next to the coordination site,^14,17,45,64^ acting as effective internal nucleophiles, the absence of this nucleophile close to the metal center of the peptoids leads to water being the sole source of external nucleophiles. Accordingly, the pH needs to be high enough to afford an adequate amount of aqueous hydroxide ions and thus to realize rapid OP hydrolysis.

PTEs with either native zinc ions or non-native metal ions showed good OP hydrolysis activities at *T* ≤ 35 °C.^26^ In contrast, our results showed that **Pep-11**–Co^2+^ membranes were able to achieve a nearly 261-fold rate enhancement when they were used at 60 °C and pH 10 (Figure 5e and Table 1, entry 25), while most natural enzymes like PTEs denatured under these conditions (Table S2).^26^ We assume that an accelerating reaction rate may involve the increasing collision of molecules, speeding up the mass transfer rate and thus improving the hydrolysis. These results showed that peptoid membrane catalysts are highly robust and can provide a much larger window for biomimetic degradation of OPs and open the way for potential practical applications.

#### Effect of Active Site Density

To investigate how the density of Nqn–Co^2+^ active sites affects the hydrolytic activity, we coassembled **Pep-5** with **Pep-1** at various molar ratios of 1:2, 1:1, and 2:1 in the presence of Co(BF_4_)_2_ to obtain peptoid–Co^2+^ membranes with various densities of Nqn–Co^2+^ active sites. Both AFM and XRD results provided strong evidence that the membrane morphology and crystallinity are not affected by the variation of peptoid ratios for cocrystallization (Figure 6a,b). We have previously used this cocrystallization approach to control the density of functional groups with peptoid membranes.^18,20^ DMNP hydrolysis results showed that peptoid–Co^2+^ membranes coassembled from **Pep-5** and **Pep-1** with a molar ratio of 1:1 showed the highest efficiency in hydrolytic activity with a *k*_cat_/*k*_uncat_ = 62.2 (Figure 6c and Table 1, entries 26–28), further reducing the molar ratio of **Pep-5** during the membrane formation and leading to the decreased hydrolytic activity. These phenomena can be attributed to the following reasons. (1) Within the crystalline **Pep-5**–Co^2+^ membranes, a high density of orderly packed Nqn–Co^2+^ active sites could limit the access of DMNP for degradation. When the density of the active sites decreased, it favored the diffusion of DMNP and its degraded product, thus leading to increased hydrolytic activity. This result is similar to previously reported nanocatalyst systems in which the dense immobilization of biocatalysts onto the surface of porous materials led to a reduction in catalytic activity.^65,66^ A further decrease of Nqn–Co^2+^ density below a 1:1 molar ratio of **Pep-5** to **Pep-1** reduced the hydrolytic activity, which could be due to the insufficient active sites available for binding with the phosphorus center of OPs for the cleavage of phosphate ester bonds.^7,10^ (2) The binding study results (Figure S19e) showed that one Co^2+^ cation coordinated with two **Pep-5** to form a complex; thus, a further decrease of **Pep-5** to have a high content of **Pep-1** (1:2 ratio) might create improper distances between Nqn, resulting in the reduced number of Nqn–Co^2+^ active sites to slow down the degradation activity.

#### Catalytic Reusability

To validate the reusability of our metal-containing peptoid membranes, we performed seven consecutive catalytic cycles using **Pep-5**–Co^2+^ membranes. After each hydrolysis cycle, the membranes were recovered by pelleting the catalyst via centrifugation and were washed several times with water prior to the next use. After seven cycles, up to 80% of the first-order rate constant was still retained, compared to the first cycle utilization (Figure 6d and Table 1, entries 6 and 29–34). In addition, AFM and TEM results showed that **Pep-5**–Co^2+^ membranes remained intact after seven-cycle hydrolysis experiments (Figures 6a and S32a). XRD results further confirmed the remaining high crystallinity and unchanged framework structure (Figure 6b). Moreover, the presence of Nqn–Co^2+^ active sites was also confirmed in the recovered membrane (Figure S32b), further supporting the successful preservation of the **Pep-5**–Co^2+^ membranes. The reduced catalytic activity could be attributed to the inevitable mass loss of the membrane sample due to repeated water washing and centrifugation. These results showed that the highly crystalline and robust **Pep-5**–Co^2+^ membranes offer great potential as stable and recyclable catalysts for efficient OP degradation.

### Proposed Mechanism

**Figure 7:**
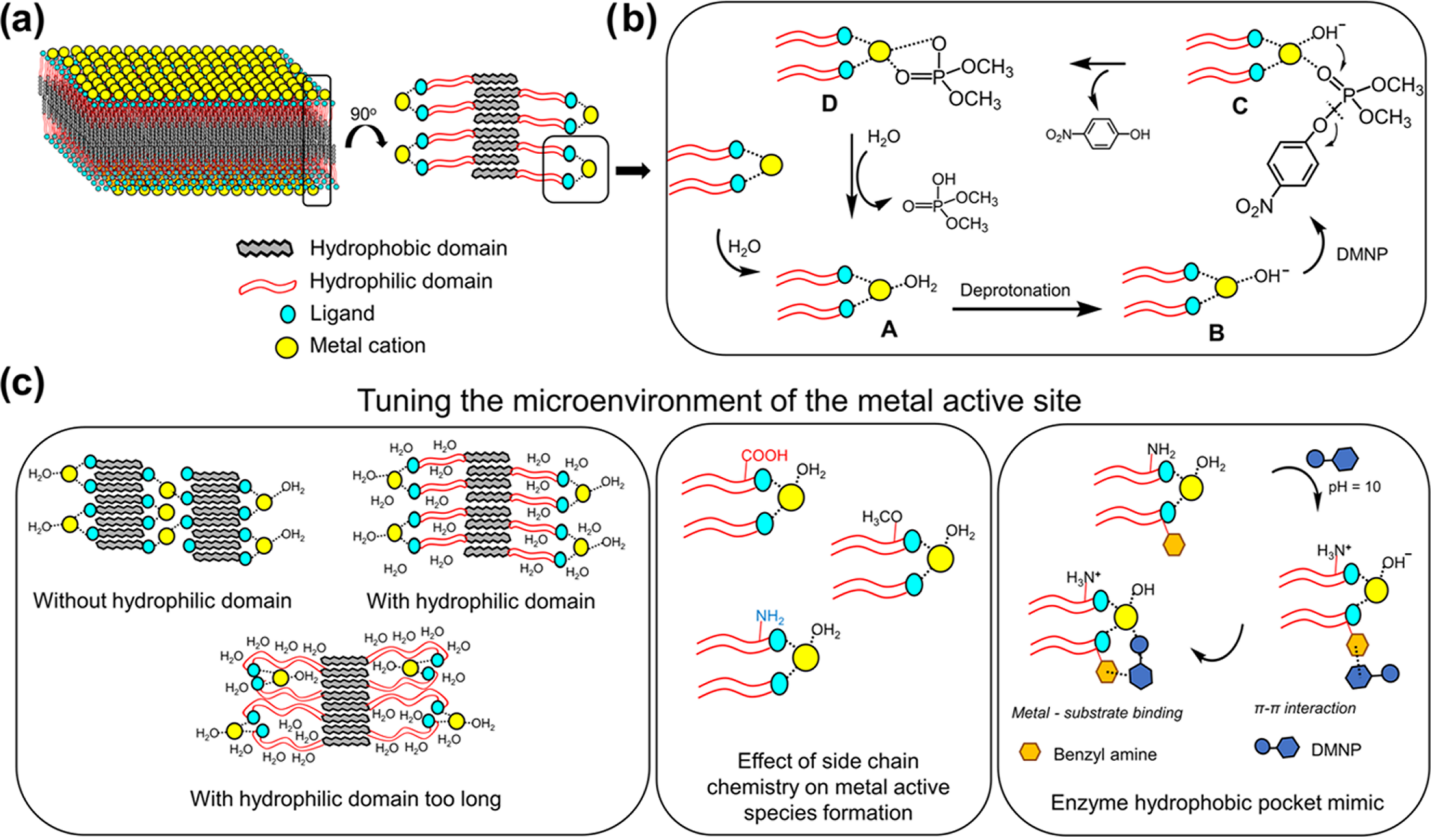
Representative scheme illustrating the tuning of metal-containing peptoid membranes for the efficient degradation of nerve agent simulants and the proposed mechanism. (a) Metal–peptoid membrane formation through the high-order packing of the hydrophobic domain. (b) Proposed model of the catalytic mechanism. (c) Tuning the microenvironment of metal-containing membranes by modifying the hydrophilic length, side-chain chemistry, and the proximity of the hydrophobic environment to the metal active site.

Our peptoid has the ability to self-assemble into a robust and crystalline nanomembrane that exposes Lewis acid metal ions on its surface, replicating the active site of PTE and mimicking the natural PTE-like microenvironment (Figure 7a). Drawing upon the well-documented effectiveness of metal-containing complexes in catalyzing the hydrolysis of organophosphates, we propose a plausible model for the hydrolysis process of DMNP, as depicted in Figure 7b.^64,67^ To initiate the process, water molecules bound to the metal ion undergo deprotonation, allowing for substrate binding. This is followed by a nucleophilic attack, resulting in the cleavage of the P–O bond. Subsequently, the attachment of a water molecule to the metal center can regenerate the active site for the next catalytic cycle.^51,68^

Given the PTE-like microenvironment of our metal-containing membranes, we possessed the capability to finely tune the catalytic activity of these membranes by systematically modifying the chemical structure of the peptoid sequences (Figure 7c). By increasing the number of –COOH groups, denoted *n*, in the hydrophilic domain, we can significantly enhance the hydrolytic performance in the following sequence: *n* = 0 < *n* = 3 < *n* = 6. As previously mentioned, the presence of water is necessary for the hydrolysis process. Hence, the presence of a hydrophilic domain is crucial, as it creates a water network around the metal center and supplies an external nucleophile for the regeneration of the metal active site. However, when the hydrophilic domain becomes too long (*n* = 9), it can lead to an excessive level of hydrophilicity and flexibility. That could reduce the effectiveness of the hydrophobic substrate DMNP to bind effectively to the active site. Therefore, achieving an optimal length of the hydrophilic domain is crucial in order to enhance the hydrolytic activity.

On the other hand, we introduced amino groups (–NH_2_) into the hydrophilic domain to emulate the presence of basic amino acid residues located adjacent to the active site of PTE. Our findings revealed that the metal-containing membranes with amino groups in the hydrophilic domain exhibited superior activity to those with –COOH and –OCH_3_ groups. We postulate that the inclusion of –NH_2_ groups can modulate the alkaline pH environment surrounding the metal active site, facilitating water deprotonation and enabling the generation of the active species metal(II)–OH^–^. Moreover, these –NH_2_ groups contribute to the regeneration of the active site after each catalytic cycle by generating hydroxyl anions.

According to reports, the active site of PTE consists of a binding pocket composed of hydrophobic residues.^57^ In line with this, we introduced Npm side chains near the metal center, which remarkably enhanced the catalytic activity. This demonstrates the essential role of having hydrophobic groups in the proximity of the active site, enabling π–π interactions with DMNP and accelerating the hydrolysis process. These findings highlight the exceptional tunability and versatility of metal-containing peptoid membranes as enzyme mimetics for the efficient degradation of organophosphates.

## Conclusions

We have successfully developed a novel type of phosphotriesterase-mimicking material using 2D crystalline peptoid nanomembranes. Our design incorporates peptoid nanomembranes with metal active sites located on their surface, enabling highly efficient degradation of organophosphates. Moreover, we tuned the hydrophilic domain and microenvironment near the ligand–metal active site by varying the peptoid chemistry to finely modulate and optimize the hydrolytic activity. Particularly, Co^2+^-containing membranes assembled from **Pep-11** with six amino groups in the hydrophilic domain exhibit a nearly 152-fold acceleration in the catalytic DMNP degradation. Further modification of **Pep-11** by installing phenyl groups directly adjacent to the metal center results in the formation of more efficient **Pep-13**–Co^2+^ membranes with an acceleration of 219-fold. These metal-containing peptoid membranes are highly stable and show efficient catalytic activity across a wide pH range, specifically pH 8–10, and at elevated temperatures, such as 60 °C. Moreover, due to their mechanical and chemical stability, these membrane materials could be easily recovered and reused multiple times, making them suitable for practical applications. Our discoveries provide an understanding of how key metalloenzyme metal centers can be mimicked and adapted to a practical material platform. This approach paves the way for future advancements in peptoid-based metalloenzyme mimetic catalysts for applications.

## Supporting Information Available

High-performance liquid chromatography-MS (HPLC-MS) of peptoid oligomers; AFM; UV–vis; XPS spectra; TEM/EDS; fluorescence images; and additional hydrolysis data (PDF)
