# Supplementary material for: Designed Metal-Containing Peptoid Membranes as Enzyme Mimetics for Catalytic Organophosphate Degradation
Source: ACS Appl Mater Interfaces. 2023 Oct 25;15(44):51191–203. doi: 10.1021/acsami.3c11816 (PMC10636725; doi:10.1021/acsami.3c11816)
Supplement: Supplementary file 1 — am3c11816_si_001.pdf [file am3c11816_si_001.pdf]

Supporting information

Designed Metal-Containing Peptoid Membranes as Enzyme Mimetics for Catalytic Organophosphate Degradation

Thi Kim Hoang Trinh,[ǂ] Tengyue Jian,[ǂ] Biao Jin,[ǂ] Dan-Thien Nguyen,[ǂ] Ronald N. Zuckermann,[£,*] Chun-Long Chen[ǂ,§,*]

[ǂ] *Physical Sciences Division, Pacific Northwest National Laboratory, Richland, WA 99352, USA*

[£]*Molecular Foundry, 1 Cyclotron Rd., Lawrence Berkeley National Laboratory, Berkeley, CA 94720, USA*

[§]*Department of Chemical Engineering, University of Washington, Washington 98195, USA*

* *Corresponding authors:* *rnzuckermann@lbl.gov* *and* *chunlong.chen@pnnl.gov*

Experimental details

Materials

All solvents were brought from Fisher or VWR and used without further purification. Millipore ultrapure water was employed throughout the experiments. Rink Amide resin (0.7-1.0 meq/g) and bromoacetic acid were purchased from Chem-Impex International, Inc. Zinc tetrafluoroborate hydrate ($Zn(BF_4)_2$) and copper(II) tetrafluoroborate hexahydrate ($Cu(BF_4)_2$) were brought from Thermo Scientific. *β*-alanine tert-butyl ester hydrochloride was purchased from Oakwood Chemical and was deprotected following the previous protocol prior to use.[1] *N,N'*-diisopropylcarbodiimide, 4-methylpiperidine, potassium carbonate (K_2CO_3), 4-bromophenyl ethylamine, *tert*-butyl-*N*-(2-aminoethyl)carbamate, 2-methoxyethyl amine and trifluoroactic acid (TFA) were purchased from Oakwood Chemical. Tris(2-aminoethyl)amine, 8-hydroxyquinoline (Nqn), N-ethylmorphine (NEM), Eriochrome Black T dye, cobalt(II) tetrafluoroborate hexahydrate ($Co(BF_4)_2$), dimethyl 4-nitrophenyl phosphonate (DMNP), and 4-(aminomethyl)pyridine (Npy) were purchased from Sigma-Aldrich. 1,4,7,10-Tetraazacyclododecane-1,4,7-triacetic acid (Do3a) was obtained from Ambeed, Inc. All amine submonomers were used as received.

Peptoid synthesis protocols

Synthesis of **Pep-1**. The synthesis of all peptoid sequences was carried out on Rink amide resins (Chem Impex) using solid-phase synthesis, following the procedures described in previous works.[1-2] In detail, the Rink amide resins (100 mg, 0.09 mmol) were first swelled in *N*,*N*-dimethylformamide (DMF) for 10 minutes. Afterward, the resins were filtered, and the Fmoc groups were removed by adding 2 mL of 20% (v/v) 4-methylpiperidine/DMF solution. The mixture was then shaken at room temperature for 40 minutes. Subsequently, the resins were drained and washed with DMF using 5 washes of 1 mL each.

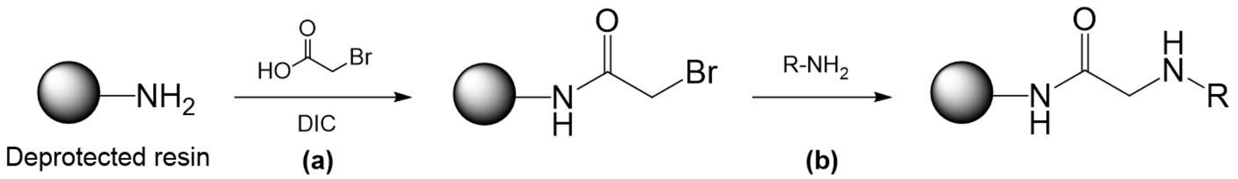

Scheme S1. General procedure illustrating the solid-phase synthesis of sequence-defined peptoids.

Afterward, the deprotected resins underwent an acylation reaction (Scheme S1a) using 1.5 mL of 0.6 M bromoacetic acid and 0.3 mL of a 50/50 (v/v) *N*,*N*-diisopropylcarbodiimide (DIC)/DMF mixture. The reaction mixture was shaken for 10 minutes at room temperature, followed by washing with DMF (5 × 1 mL). Nucleophilic displacement of bromide with the submonomers (Scheme S1b) was achieved by adding 1.5 mL of a 0.6 M primary amine solution in *N*-methyl-2-pyrrolidone (NMP) and agitating for 10 minutes at room temperature. The solution was then filtered, and the resins were washed with DMF (5 × 1 mL). The acylation and displacement reactions with appropriate primary amines [such as 4-bromophenethylamine (Nbrpe) or *β*-alanine tert-butyl ester (Nce)] were repeated until the desired target peptoid sequence was obtained.

Synthesis of **Pep-2**. **Pep-2** was synthesized through the functionalization of **Pep-1** using the submonomer procedure. Typically, **Pep-1** (0.09 mmol, 1 equiv.) was mixed with 1.5 mL of 0.6 M chloroacetic acid and 0.3 mL of a 50/50 (v/v) DIC/DMF mixture for 10 minutes. The supernatant was then removed, and the resin was washed with DMF (5 × 1 mL). Next, 1.5 mL of 0.6 M 4-(aminomethyl)pyridine (Npy) in NMP was added to the resin. The mixture was stirred for 2 hours at 40°C, filtered, and washed with DMF (5 × 1 mL).

Synthesis of **Pep-3**. **Pep-3** was synthesized through the functionalization of **Pep-2** using the submonomer procedure. Typically, **Pep-2** (0.09 mmol, 1 equiv.) was mixed with 1.5 mL of 0.6 M chloroacetic acid and 0.3 mL of a 50/50 (v/v) DIC/DMF mixture for 10 minutes. After removing the supernatant, the resin was washed with DMF (5 × 1 mL). Next, 1.5 mL of 0.6 M Npy in NMP was added to the resin. The mixture was stirred for 2 hours at 40°C, filtered, and washed with DMF (5 × 1 mL).

Synthesis of **Pep-4**. **Pep-4** was synthesized through the functionalization of **Pep-1** using the submonomer procedure. Typically, **Pep-1** (0.09 mmol, 1 equiv.) was mixed with 1.5 mL of 0.6 M bromoacetic acid and 0.3 mL of a 50/50 (v/v) DIC/DMF mixture for 10 minutes. After removing the supernatant, the resin was washed with DMF (5 × 1 mL). Subsequently, 1.5 mL of 0.6 M 8-hydroxyquinoline (Nqn) in NMP, along with 100 mg of K_2CO_3, were added to the resin. The mixture was stirred overnight at room temperature, filtered, and washed with DMF (5 × 1 mL).

Syntheses of **Pep-5, Pep-7, Pep-8** and **Pep-9**. **Pep-5**, **Pep-7, Pep-8** and **Pep-9** were synthesized through the functionalization of **Pep-1** via the submonomer procedure. Typically, **Pep-1** (0.09 mmol, 1 equiv.) was mixed with 1.5 mL of 0.6 M bromoacetic acid and 0.3 mL of 50/50 (v/v) DIC/ DMF for 10 min before removing the supernatant and washing with DMF (5 × 1 mL). Subsequently, 1.5 mL of 0.6 M of tris(2-aminoethyl)amine in NMP was added to the above resin, and the mixture was stirred overnight at room temperature, filtered, and washed with DMF (5 × 1 mL). One more acylation cycle was performed using 3 mL of 0.6 M bromoacetic acid and 0.6 mL of 50/50 (v/v) DIC/ DMF. Afterward, 3 mL of 0.6 M Nqn in NMP and 200 mg K_2CO_3 were added to the above resin, and the mixture was stirred overnight at room temperature, filtered, and washed with DMF (5 × 1 mL).

Synthesis of **Pep-6**. **Pep-6** was synthesized through the functionalization of **Pep-1** using the submonomer procedure. Typically, **Pep-1** (0.09 mmol, 1 equiv.) was mixed with 1.5 mL of 0.6 M bromoacetic acid and 0.3 mL of a 50/50 (v/v) DIC/DMF mixture for 10 minutes. After removing the supernatant, the resin was washed with DMF (5 × 1 mL). Subsequently, a mixture containing 1,4,7,10-tetraazacyclododecane-1,4,7-triacetic acid trisodium salt (Do3a) (231.3 mg, 0.45 mmol, 5 equiv.), K_2CO_3 (24.9 mg, 0.18 mmol, 2 equiv.), and tetra-N-butylammonium iodide (49.9 mg, 0.135 mmol, 1.5 equiv.) in 3 mL of DMF was added to the resin. The reaction mixture was stirred overnight at room temperature, filtered, and washed with DMF (5 × 1 mL.

Synthesis of **Pep-10**. To synthesize the first 12 units of **Pep-10**, the protocol used for **Pep-1** was repeated. However, in the later acylation steps, 2-methoxyethyl amine (Nome) was used instead of Nce. After the completion of these steps, the resulting intermediate was further functionalized with Nqn using a protocol similar to that of **Pep-5**.

Synthesis of **Pep-11**. To synthesize the first 12 units of **Pep-11**, the protocol used for **Pep-1** was repeated. However, in the later acylation steps, tert-butyl (2-aminoethyl)carbamate (Nae) was used instead of Nce. After completing these steps, the resulting intermediate was further functionalized with Nqn using a protocol similar to that of **Pep-5**.

Synthesis of **Pep-12** and **Pep-13**. To synthesize the first 12 units of **Pep-12** and **Pep-13**, the protocol of **Pep-1** was repeated. However, the Nome and Nae were used instead of Nce in the later acylation steps. Then, it was further functionalized with Nqn by using a similar protocol of **Pep-5**.

Synthesis of **Pep-14**. To synthesize the first 12 units of **Pep-14**, the synthetic protocol of **Pep-1** was repeated. However, the Nae was used instead of Nce in the six later acylation steps. Then, it was mixed with 1.5 mL of 0.6 M bromoacetic acid and 0.3 mL of 50/50 (v/v) DIC/DMF for 10 min before removing the supernatant and washing with DMF (5 × 1 mL). Subsequently, 1.5 mL of 0.6 M of tris(2-aminoethyl)amine in NMP was added to the above resin, and the mixture was stirred overnight at room temperature, filtered, and washed with DMF (5 × 1 mL). Furthermore, an acylation cycle was performed using 1.5 mL of 0.6 M bromoacetic acid and 0.6 mL of 50/50 (v/v) DIC/DMF. Nucleophilic displacement of bromide with the submonomers occurred by adding 1.5 mL of 0.6 M benzylamine solution in NMP, followed by agitating for 10 min at room temperature. The solution was filtered off, and the resins were washed with DMF (5 × 1 mL). One more acylation cycle was performed using 3 mL of 0.6 M bromoacetic acid and 0.6 mL of 50/50 (v/v) DIC/ DMF. Finally, 3 mL of 0.6 M Nqn in NMP and 200 mg K_2CO_3 were added to the above resin, and the mixture was stirred overnight at room temperature, filtered, and washed with DMF (5 × 1 mL).

Synthesis of **Pep-15**. To synthesize the first 12 units of **Pep-15**, the synthetic protocol of **Pep-1** was repeated. However, the Nae was used instead of Nce in the six later acylation steps. Then, it was mixed with 1.5 mL of 0.6 M bromoacetic acid and 0.3 mL of 50/50 (v/v) DIC/DMF for 10 min before removing the supernatant and washing with DMF (5 × 1 mL). Subsequently, 1.5 mL of 0.6 M of tris(2-aminoethyl)amine in NMP was added to the above resin, and the mixture was stirred overnight at room temperature, filtered, and washed with DMF (5 × 1 mL). Furthermore, an acylation cycle was performed using 1.5 mL of 0.6 M bromoacetic acid and 0.6 mL of 50/50 (v/v) DIC/DMF. Nucleophilic displacement of bromide with the submonomers occurred by adding 1.5 mL of 0.6 M isopropyl amine solution in NMP, followed by agitating for 10 min at room temperature. The solution was filtered off, and the resins were washed with DMF (5 × 1 mL). One more acylation cycle was performed using 3 mL of 0.6 M bromoacetic

acid and 0.6 mL of 50/50 (v/v) DIC/ DMF. Finally, 3 mL of 0.6 M Nqn in NMP and 200 mg K_2CO_3 were added to the above resin, and the mixture was stirred overnight at room temperature, filtered, and washed with DMF (5 × 1 mL).

Purification and mass spectrometry analysis.

The cleavage of peptoids from the resins was carried out by treating the bead resins with 3 mL of 95/5 (v/v) trifluoroacetic acid (TFA)/H_2O for 30 minutes with agitation. The solution was collected and the TFA solution was evaporated under reduced pressure at 36 °C. The crude peptoid product was dissolved in 80/20 (v/v) acetonitrile/ H_2O and purified by reverse-phase high-performance liquid chromatography (HPLC, Water 1525) set up with an XBridge™ Prep C18 OBDTM column, 10 µm, 19 mm×100 mm, using a linear gradient of 45 – 55 % (for **Pep-2**, **Pep-5**) or 50 – 70% (for the other peptoids) acetonitrile in water with 0.1% TFA. Mass spectrometry characterization of purified peptoids was conducted following our previous work.[2]

Evaluation of metal-peptoid binding

UV-vis titration. The binding of metal and peptoid was examined via UV-vis titration in acetonitrile solvent. After collecting the blank spectrum of acetonitrile in the 250 – 700 nm range, 100 uL of peptoids (1 mM stock solution) was diluted in 900 uL of acetonitrile to attain a final concentration of 0.1 mM. Each peptoid was titrated separately to gain the absorbance spectrum of individual complexes by adding 1 uL of $Co(BF_4)_2$ solution. Extracting the change in absorbance at a specific wavelength provides the binding ratio between metal and peptoid.

Quantitative metal-peptoid binding efficiency. The amount of metal held on the peptoid membranes was systematically determined through the colorimetry titration procedure using Eriochrome Black T (EBT) as a titrant.[3-4] To assemble the metal-peptoid membrane, water was added to reach a final volume of 600 µL, and then the solution was centrifuged at 8,000 × g for 10 min. Subsequently, 400 µL of supernatant was collected, and fresh 400 µL of water was added to the peptoid solution again to perform 2nd washing cycle. This washing process was repeated until no metal bleaching was detected. To determine the metal residence

in the collected supernatant, 10 μL of supernatant was added to 610 μL of EBT (70 mM, pH=10). The change in the absorbance of EBT was monitored by using a UV/vis spectrometer. A similar procedure was conducted with the fresh metal salt solutions to establish the calibration curve (Figure S20).

Catalytic activity test of metal-peptoid nanomembranes toward DMNP hydrolysis

The catalytic activity was carried out in a Tecan Safire 2 microplate reader. Each well was prepared with 180 μL of a total volume containing: 153 uL buffer ([N-ethylmorpholine]= 20 mM, pH=10), 22.5 μL of catalyst (1 mM of peptoid powder, metal salts or self-assembled peptoid premixed in water and sonicated before using) and 4.5 μL of DMNP ([DMNP= 10 mM in methanol). Upon adding the substrate, the 96-well plate was immediately put into the reader to monitor the change in absorbance at 400 nm. The conversion was calculated based on an established calibration curve with various given amounts of 4-nitrophenol in NEM buffer (Figure S26b). Reported activity is the average value for three replicates with background subtraction.

A general protocol for recycle peptoid membranes

After each catalytic cycle, the reaction mixture was transferred into a 1.5 mL Eppendorf tube. The peptoids were then collected by centrifuging at 10,000 × *g* for 10 minutes. Subsequently, the supernatant was gently removed using a pipette, and the membrane was resuspended in fresh water. This process of centrifugation, pelleting the membrane, and resuspension in water was repeated twice. Prior to hydrolysis, the recovered peptoid membranes were resuspended in NEM buffer for a minimum of 2 hours.

Characterization

Atomic force microscopy (AFM) characterization. Ex situ AFM analysis was carried out on a Brucker MultiMode 8 by using ScanAsyst mode at room temperature. For sample preparation, 1.5 μL of the self-assembled peptoid was diluted 40 × with deionized water and deposited onto a mica substrate. After 5 min, Whatman filter paper was applied to remove the solution, and then the dried mica substrate was further dried under N_2 flow). The AFM probe consisted of silicon tips on silicon cantilevers (NCHV probes, k = 42 N/m, tip radius).

Transmission electron microscopy (TEM). TEM images were taken from the samples on a 3-mm diameter copper grid coated with carbon film using the FEI Tecnai G2 transmission electron microscope with an accelerating voltage of 200 kV and ETEM (FEI) operated at 200 kV.

X-ray diffraction analysis (XRD). All characterization was carried out following the procedure described previously.[1] Typically, powder XRD data were gathered on a multiple wavelength anomalous diffraction and monochromatic macromolecular crystallography beamline, 8.3.1 (possessing a 5 T single pole superbend source with an energy range of 5–17 keV) at the Advanced Light Source located at Lawrence Berkeley National Laboratory. All XRD data were collected with a 3 × 3 CCD array (ADSC Q315r) detector at a wavelength of 1.11583 Å. Datasets were collected with the detector 200 mm from the sample. For XRD sample preparations, samples of peptoid assemblies were loaded onto a Kapton mesh (MiTeGen) and dried for XRD measurements. All XRD Data were analyzed using custom Python scripts.

Fluorescence analysis of assembled nanomembranes. The peptoid assemblies were labeled with Nile red dye using a previously established protocol.[5] The labeled membranes were then visualized using the Zeiss LSM 710 confocal microscope system, which is equipped with a Zeiss AXIO Observer Z1 inverted microscope stand. The microscope system utilizes transmitted (HAL), UV (HBO), and laser illumination sources. For imaging, the samples were excited at 561 nm and images were collected at a magnification of 20× and 40×.

X-ray photoelectron spectroscopy (XPS) analysis. XPS analysis was performed using a Kratos Axis Ultra DLD spectrometer, which consists of an Al Kα monochromatic X-ray source (1486.6 eV) and a high-resolution spherical mirror analyzer. X-ray source was operated at 150 W power and the emitted photoelectrons were collected at the analyzer entrance slit normal to the sample surface. The high-resolution spectra were collected at a pass energy of 40 eV with a step sized of 0.1 eV. The Kratos charge neutralizer system was used for all analyses with charge neutralization being monitored using the C 1s signal. XPS data were analyzed using CasaXPS software. All the XPS peaks were charge referenced to C 1s signal (C-C/C-H) at 285 eV.

Additional figures

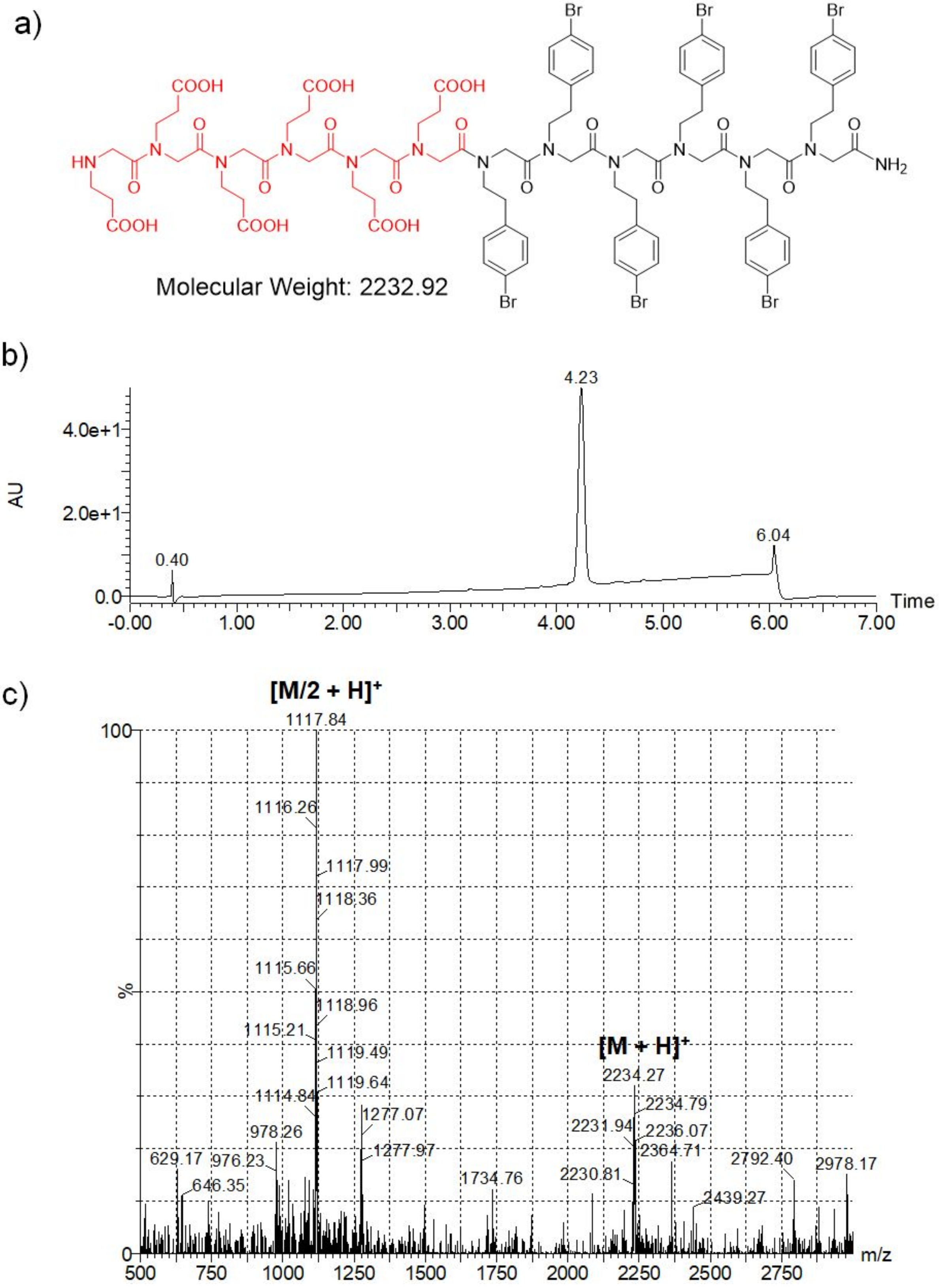

Figure S1. UPLC characterization of **Pep-1**. a) Chemical structure. b) LC-MS chromatogram with the gradient of 5 - 95% CH_3CN in H_2O. c) ESI^+ ionization pattern.

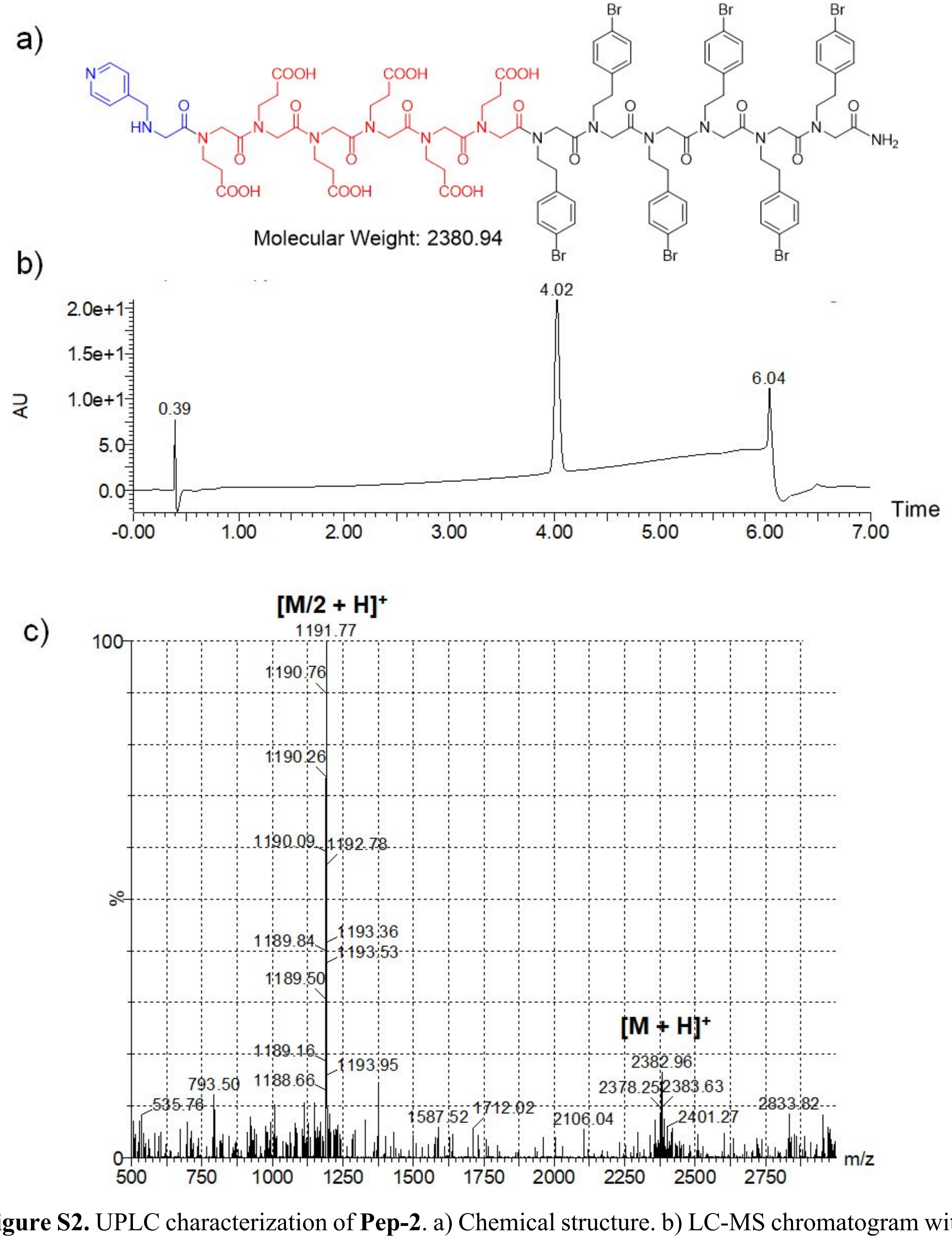

Figure S2. UPLC characterization of **Pep-2**. a) Chemical structure. b) LC-MS chromatogram with the gradient of 5 - 95% CH_3CN in H_2O. c) ESI^+ ionization pattern.

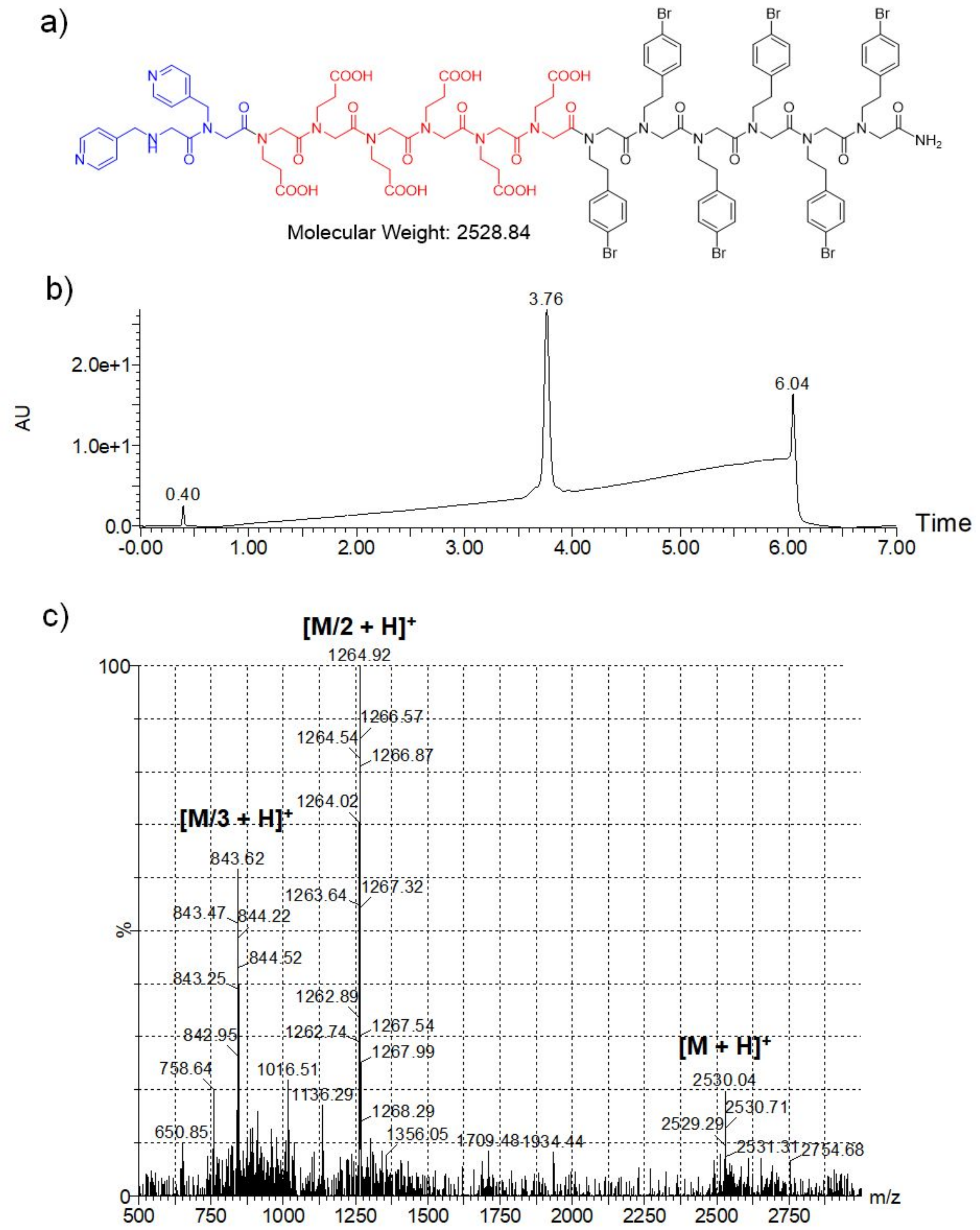

Figure S3. UPLC characterization of **Pep-3**. a) Chemical structure. b) LC-MS chromatogram with the gradient of 5 - 95% CH_3CN in H_2O. c) ESI^+ ionization pattern.

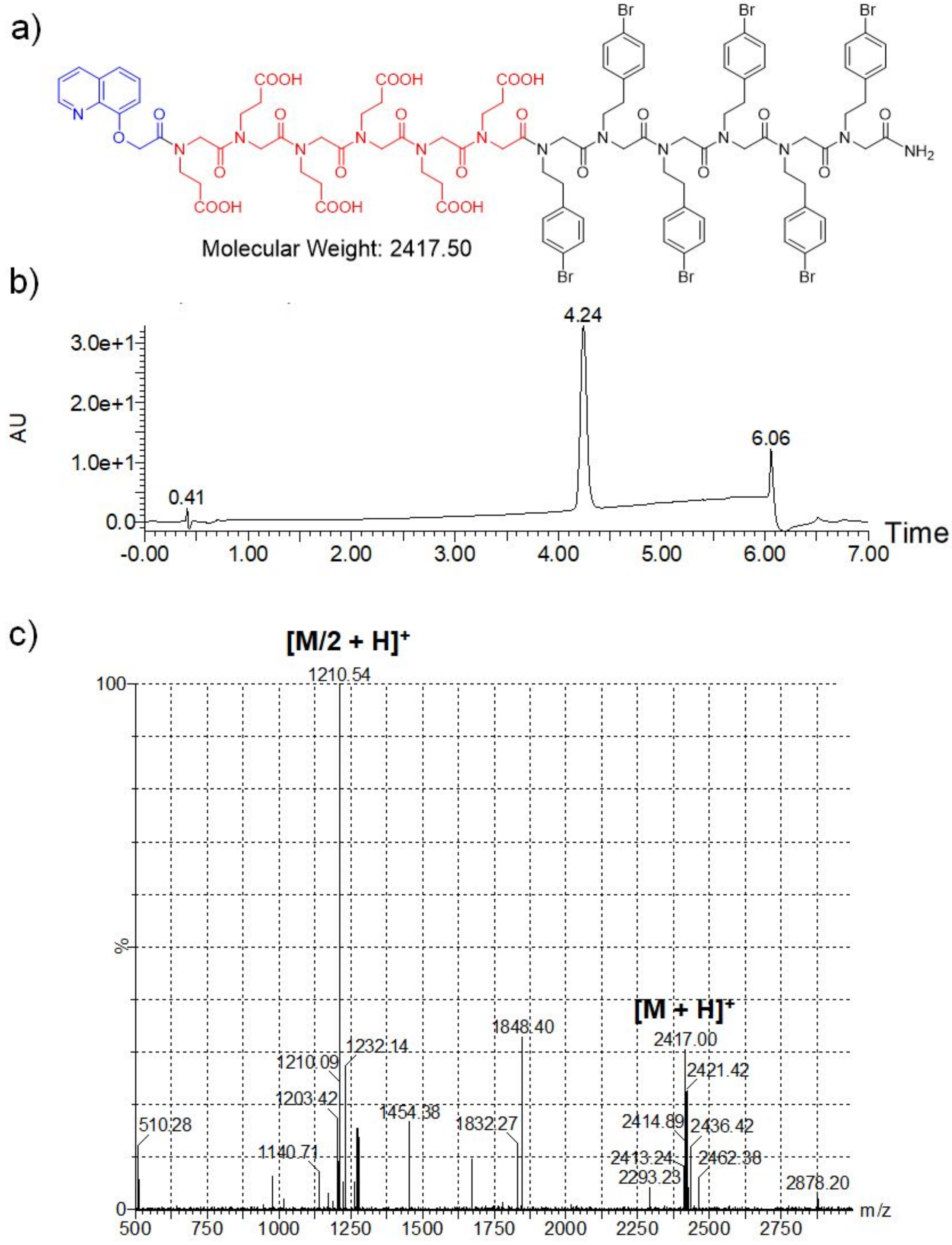

Figure S4. UPLC characterization of **Pep-4**. a) Chemical structure. b) LC-MS chromatogram with the gradient of 5 - 95% CH_3CN in H_2O. c) ESI^+ ionization pattern.

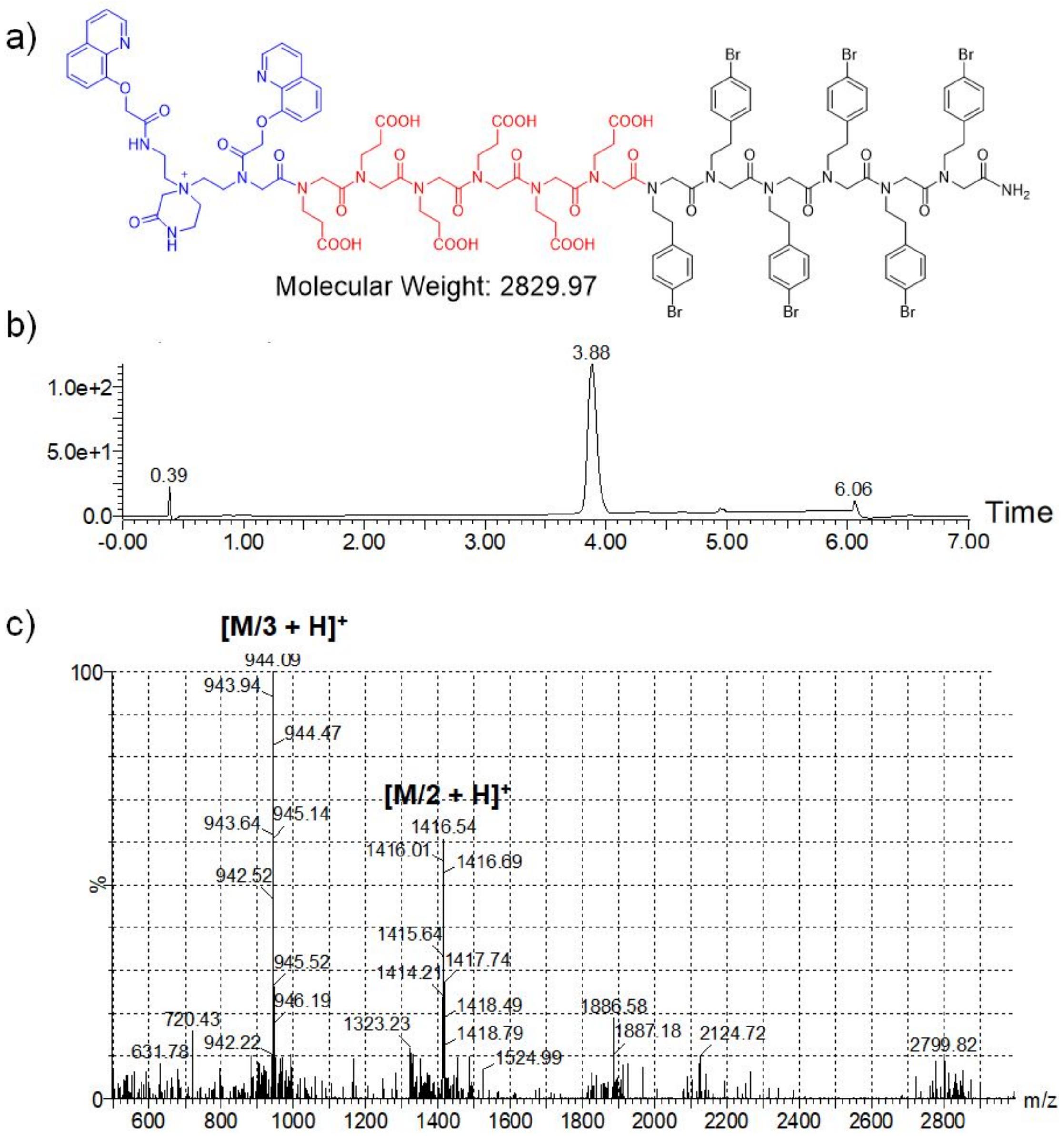

Figure S5. UPLC characterization of **Pep-5**. a) Chemical structure. b) LC-MS chromatogram with the gradient of 5 - 95% CH_3CN in H_2O. c) ESI^+ ionization pattern.

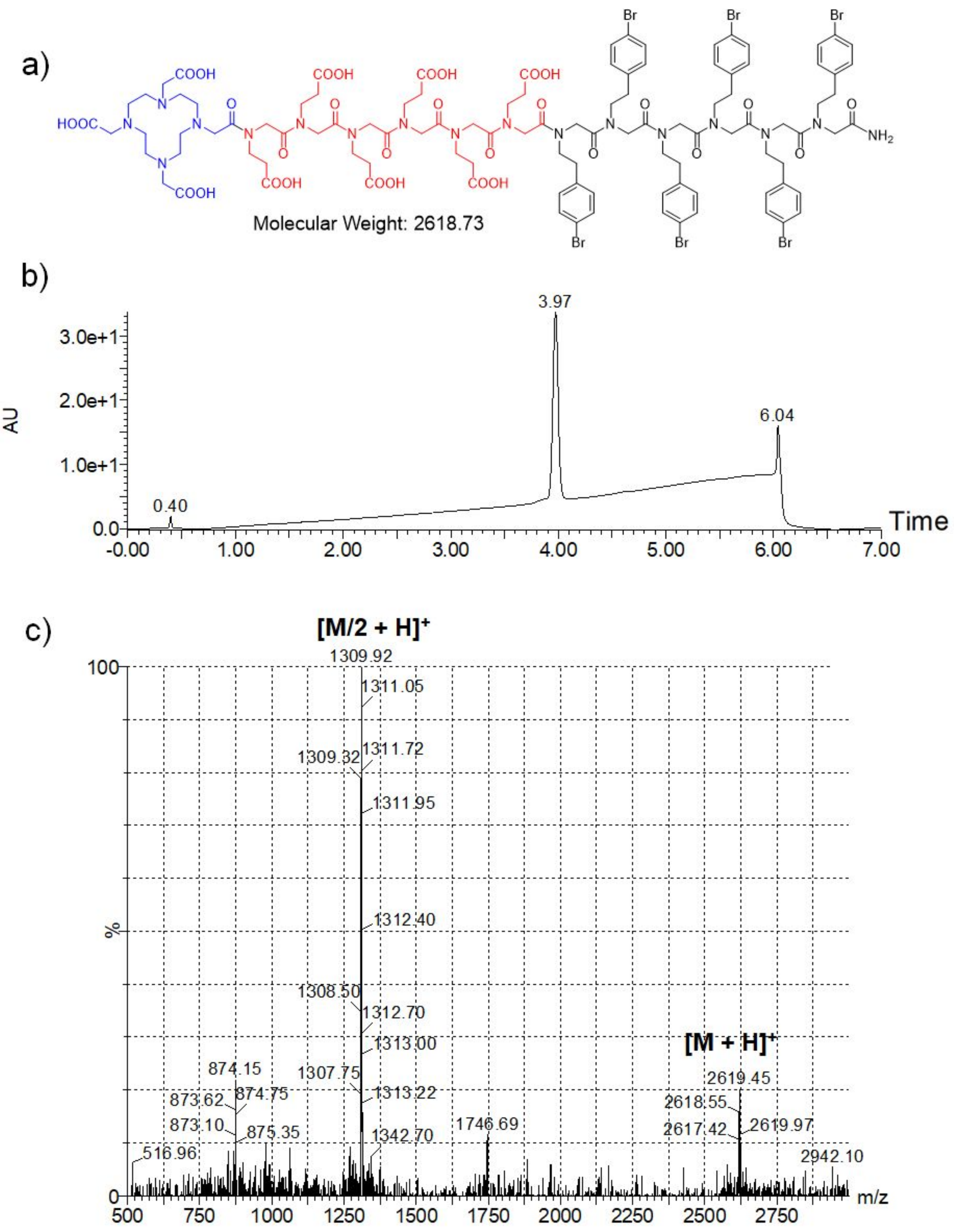

Figure S6. UPLC characterization of **Pep-6**. a) Chemical structure. b) LC-MS chromatogram with the gradient of 5 - 95% CH_3CN in H_2O. c) ESI^+ ionization pattern.

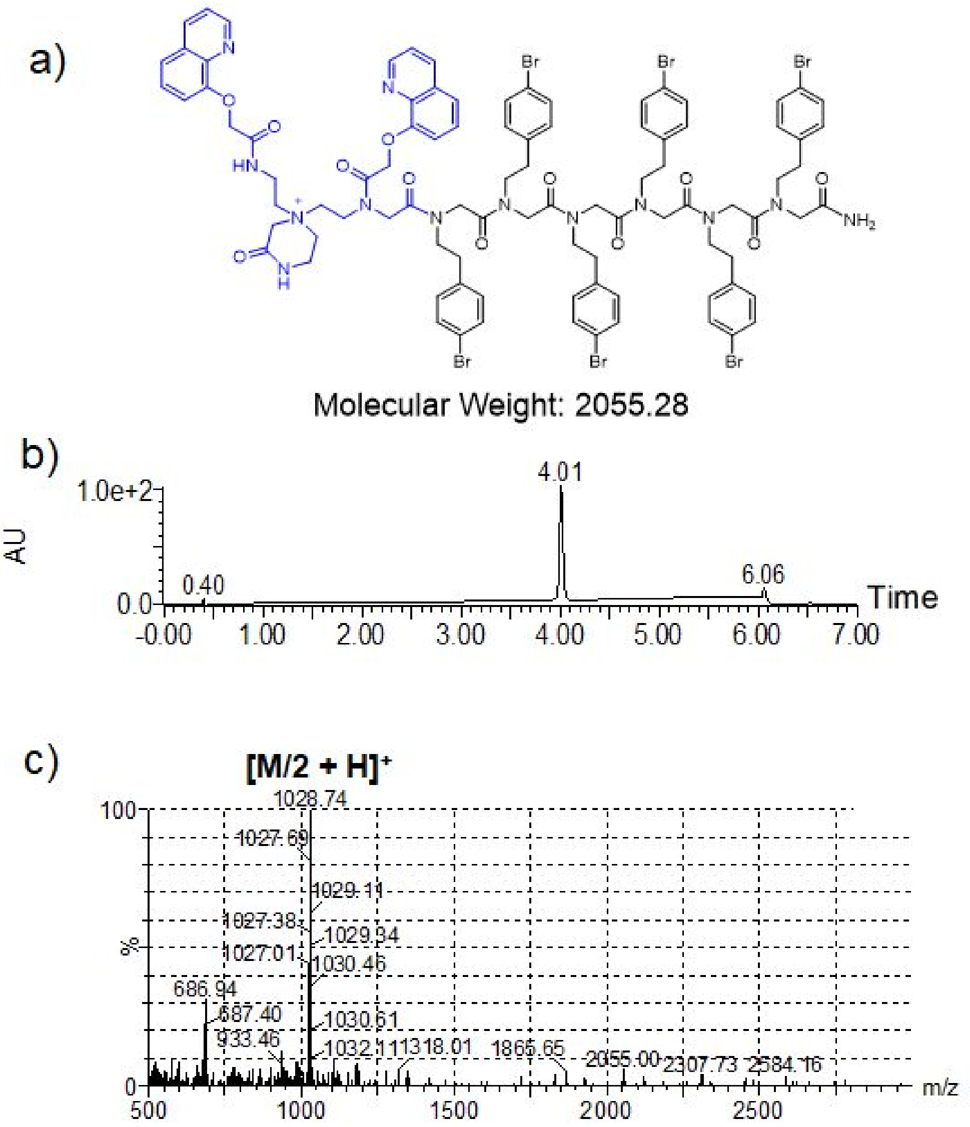

Figure S7. UPLC characterization of **Pep-7**. a) Chemical structure. b) LC-MS chromatogram with the gradient of 5 - 95% CH_3CN in H_2O. c) ESI^+ ionization pattern.

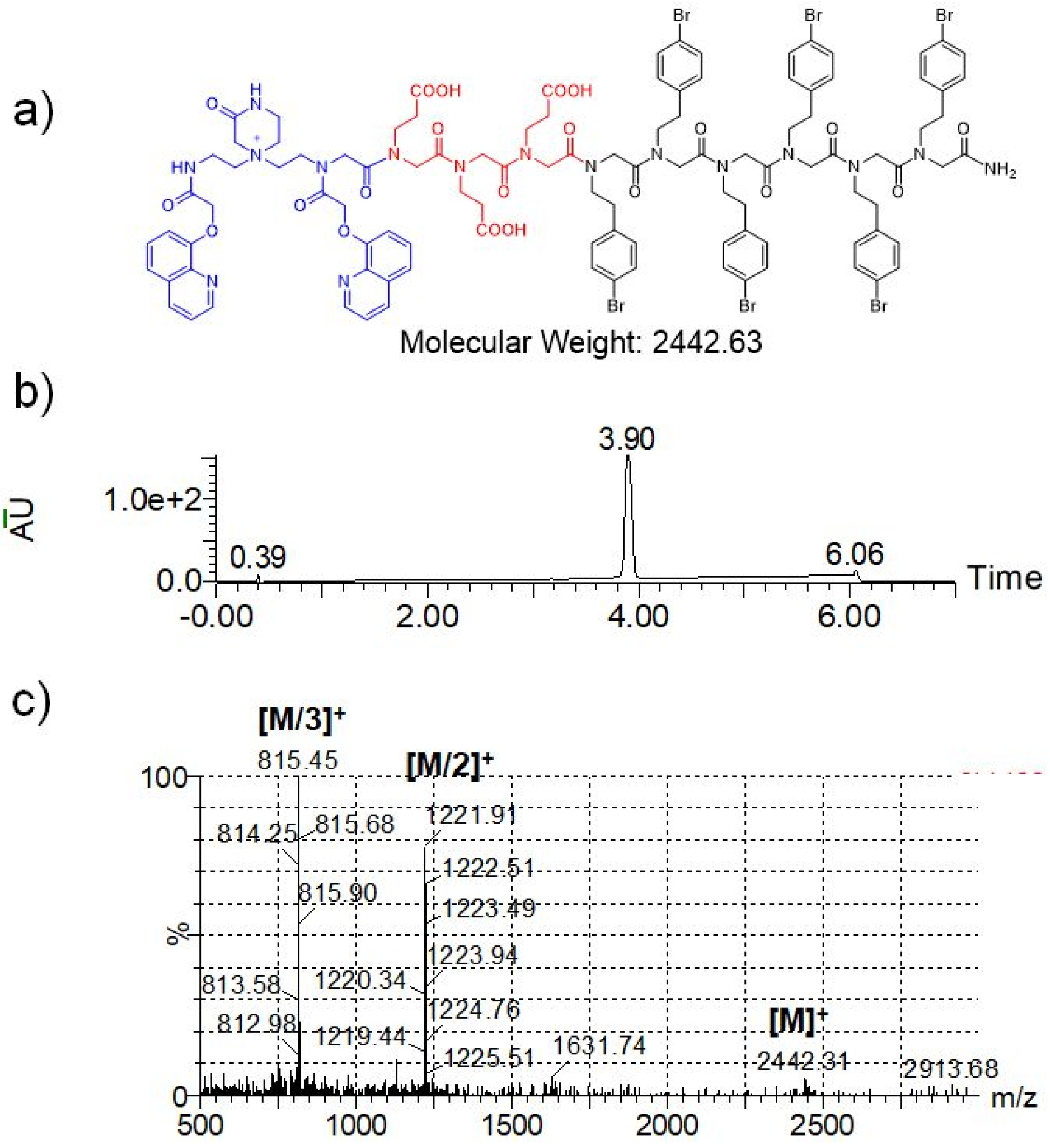

Figure S8. UPLC characterization of **Pep-8**. a) Chemical structure. b) LC-MS chromatogram with the gradient of 5 - 95% CH_3CN in H_2O. c) ESI^+ ionization pattern.

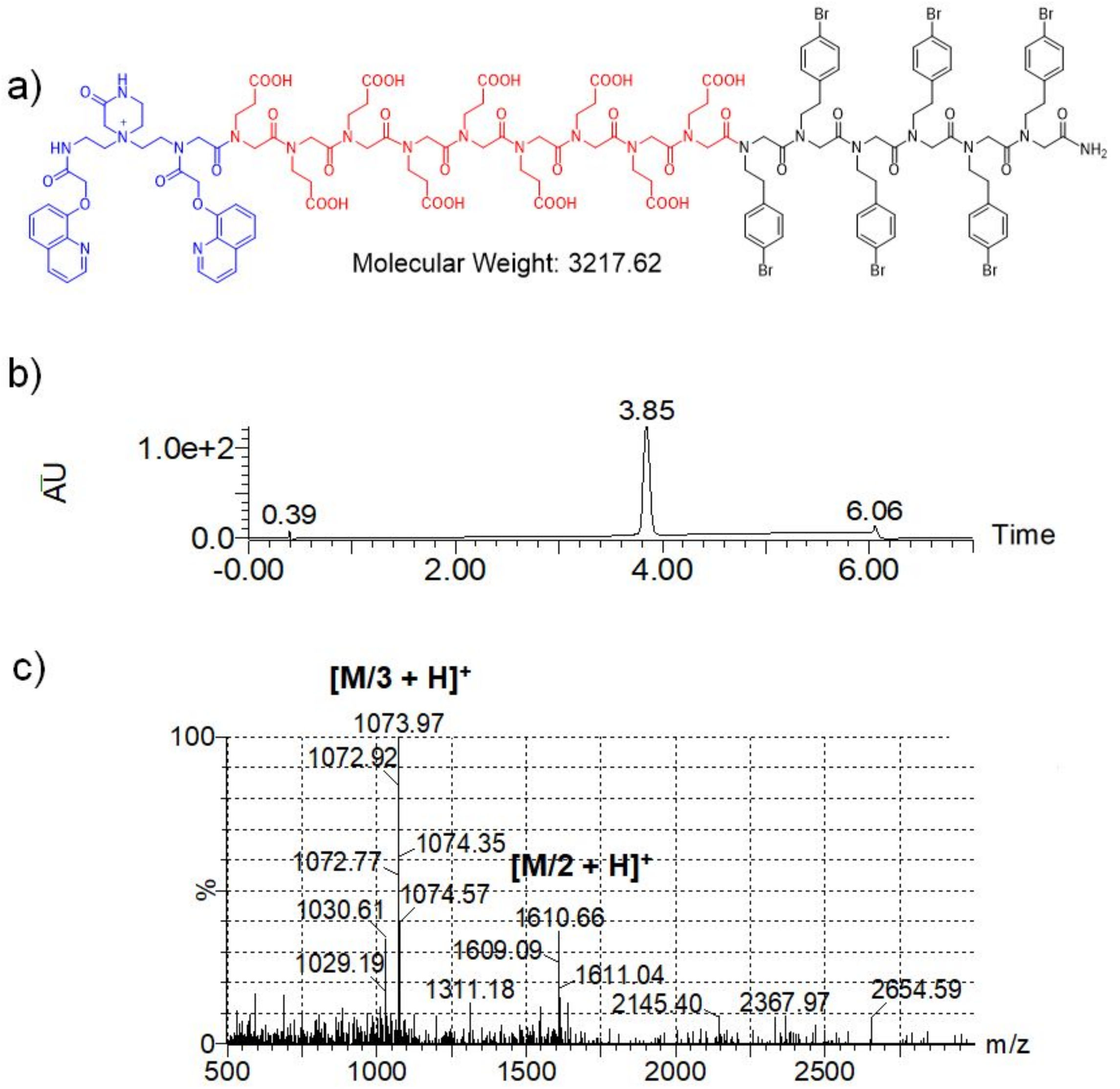

Figure S9. UPLC characterization of **Pep-9**. a) Chemical structure. b) LC-MS chromatogram with the gradient of 5 - 95% CH_3CN in H_2O. c) ESI^+ ionization pattern.

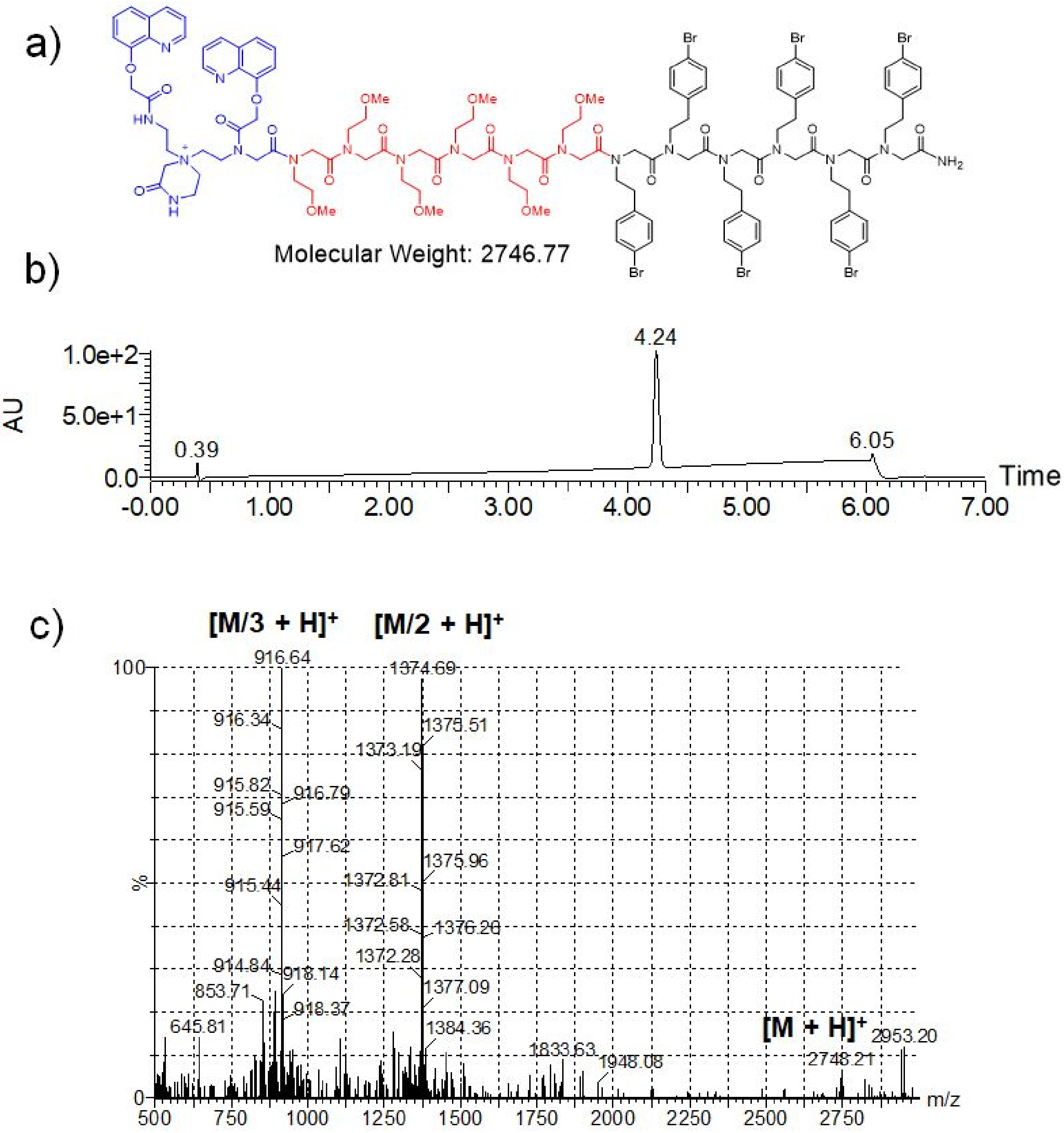

Figure S10. UPLC characterization of **Pep-10**. a) Chemical structure. b) LC-MS chromatogram with the gradient of 5 - 95% CH_3CN in H_2O. c) ESI^+ ionization pattern.

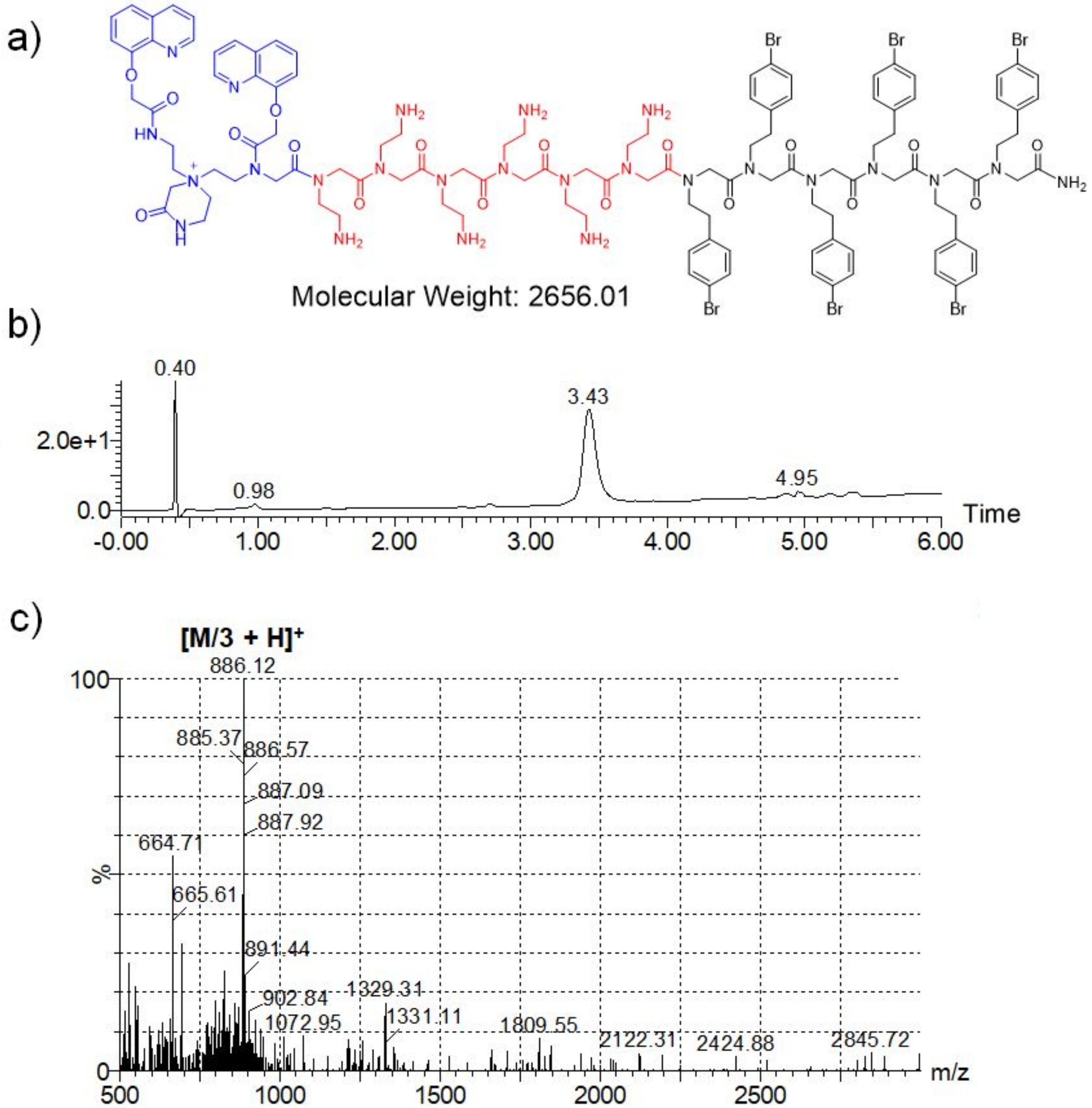

Figure S11. UPLC characterization of **Pep-11**. a) Chemical structure. b) LC-MS chromatogram with the gradient of 5 - 95% CH_3CN in H_2O. c) ESI^+ ionization pattern.

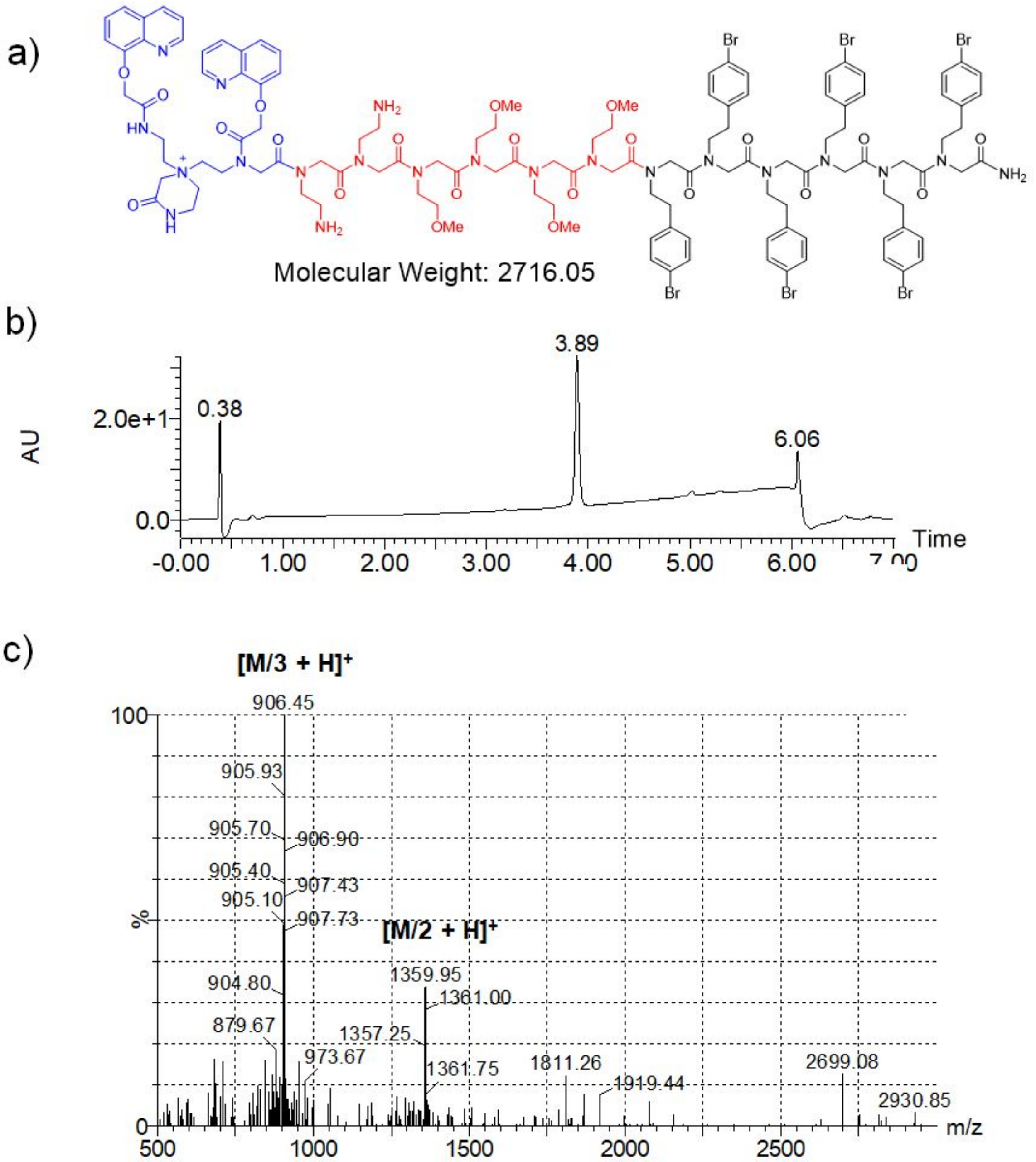

Figure S12. UPLC characterization of **Pep-12**. a) Chemical structure. b) LC-MS chromatogram with the gradient of 5 - 95% CH_3CN in H_2O. c) ESI^+ ionization pattern.

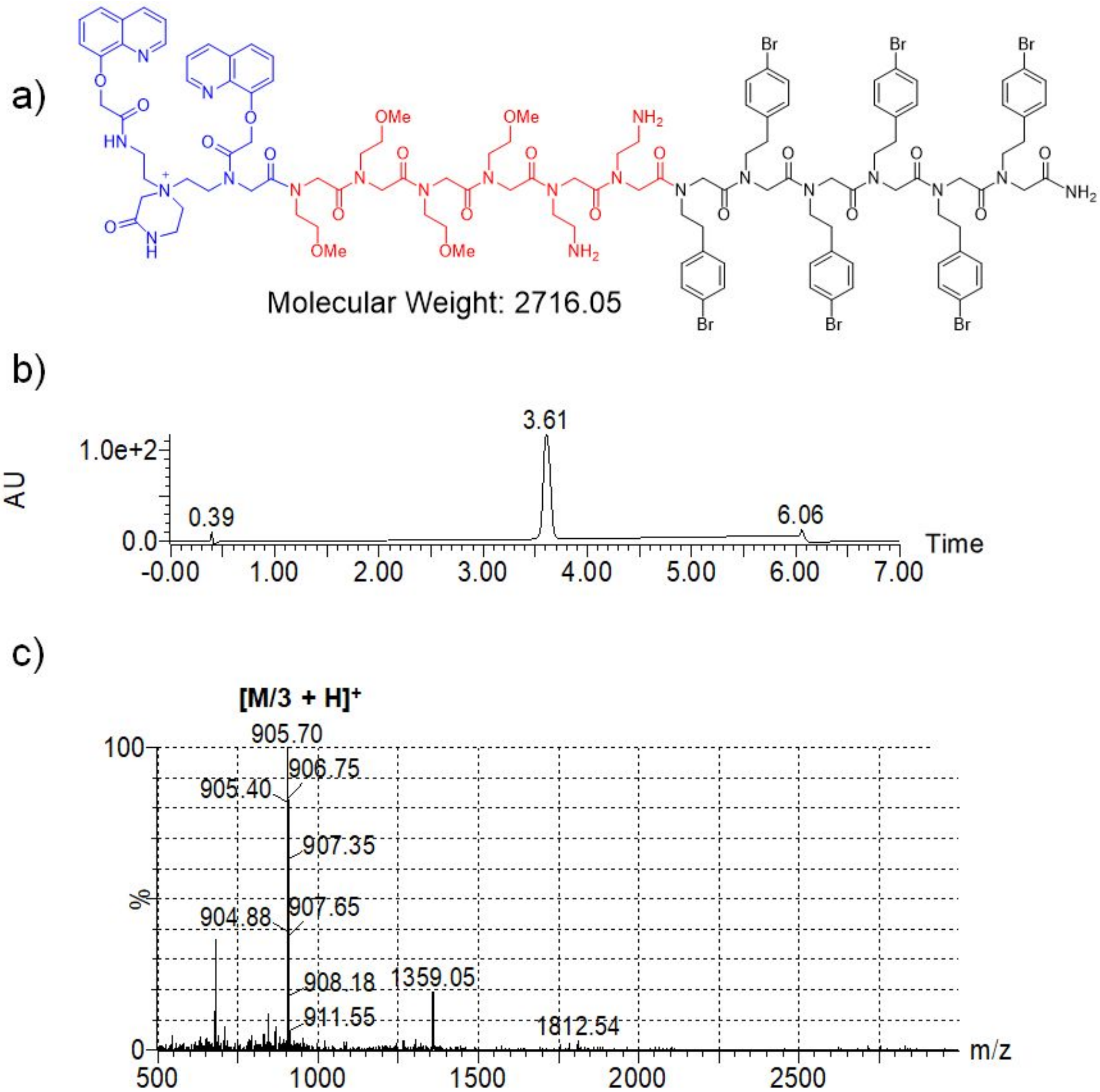

Figure S13. UPLC characterization of **Pep-13**. a) Chemical structure. b) LC-MS chromatogram with the gradient of 5 - 95% CH_3CN in H_2O. c) ESI^+ ionization pattern.

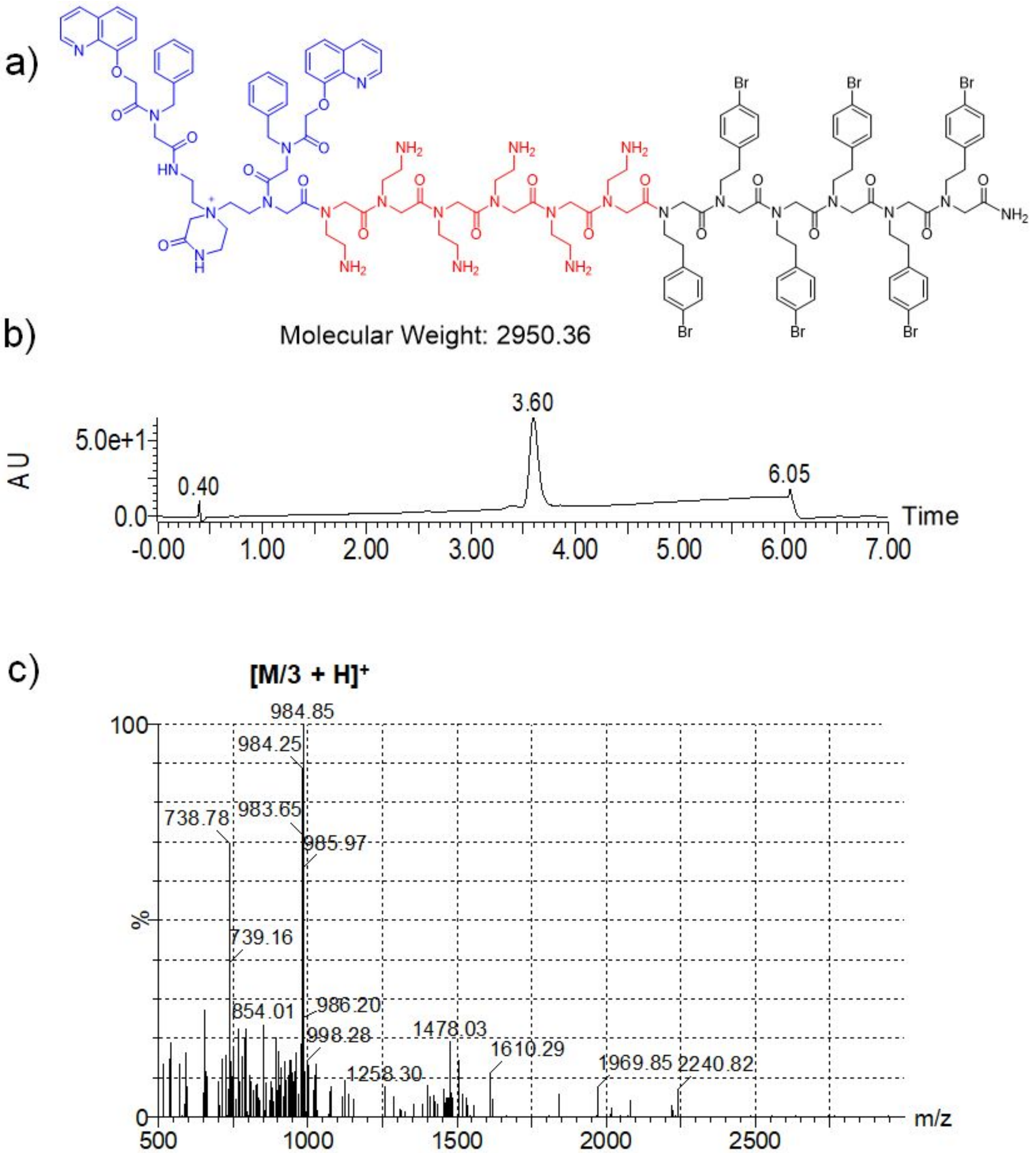

Figure S14. UPLC characterization of **Pep-14**. a) Chemical structure. b) LC-MS chromatogram with the gradient of 5 - 95% CH_3CN in H_2O. c) ESI^+ ionization pattern.

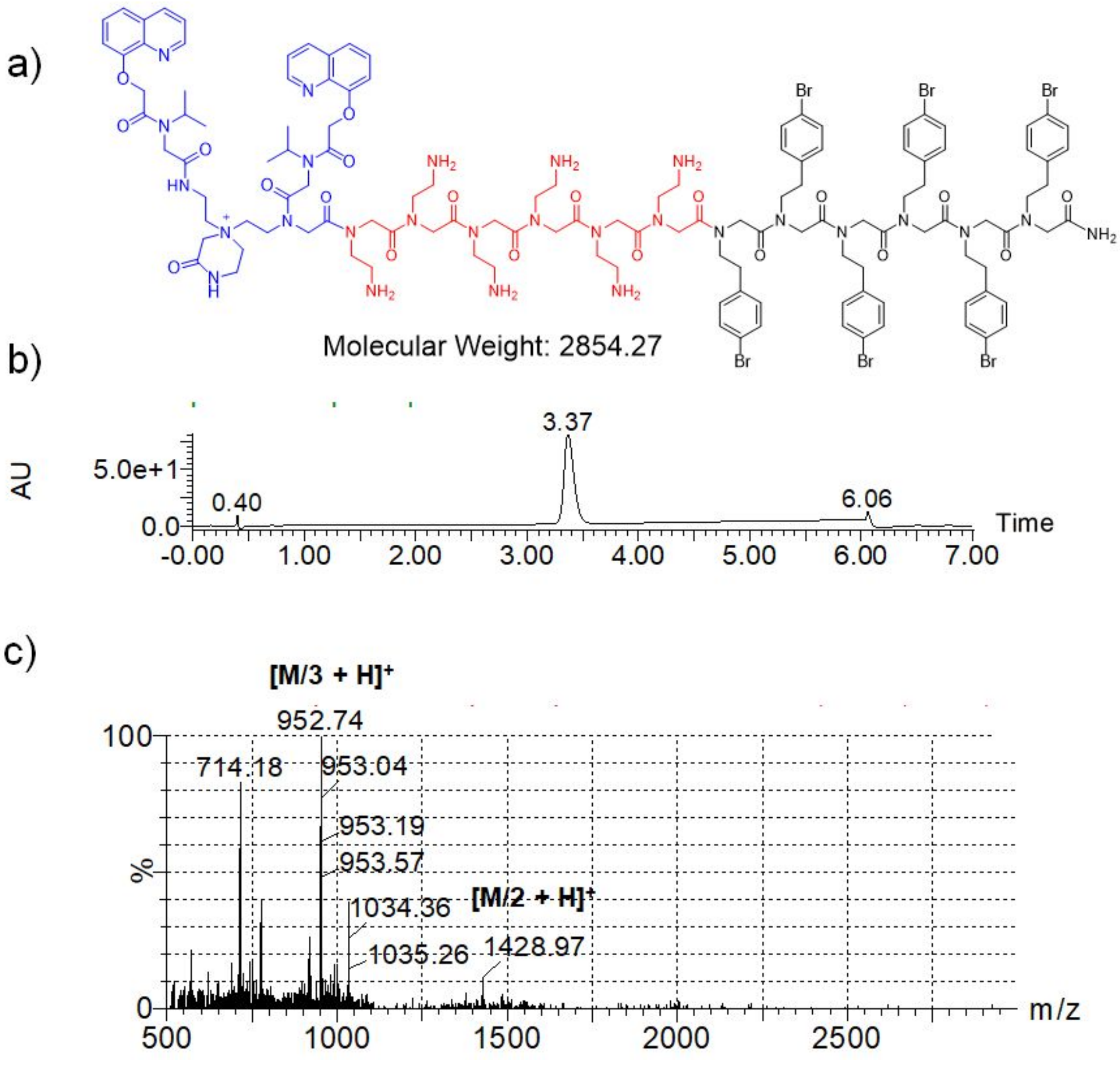

Figure S15. UPLC characterization of **Pep-15**. a) Chemical structure. b) LC-MS chromatogram with the gradient of 5 - 95% CH_3CN in H_2O. c) ESI^+ ionization pattern.

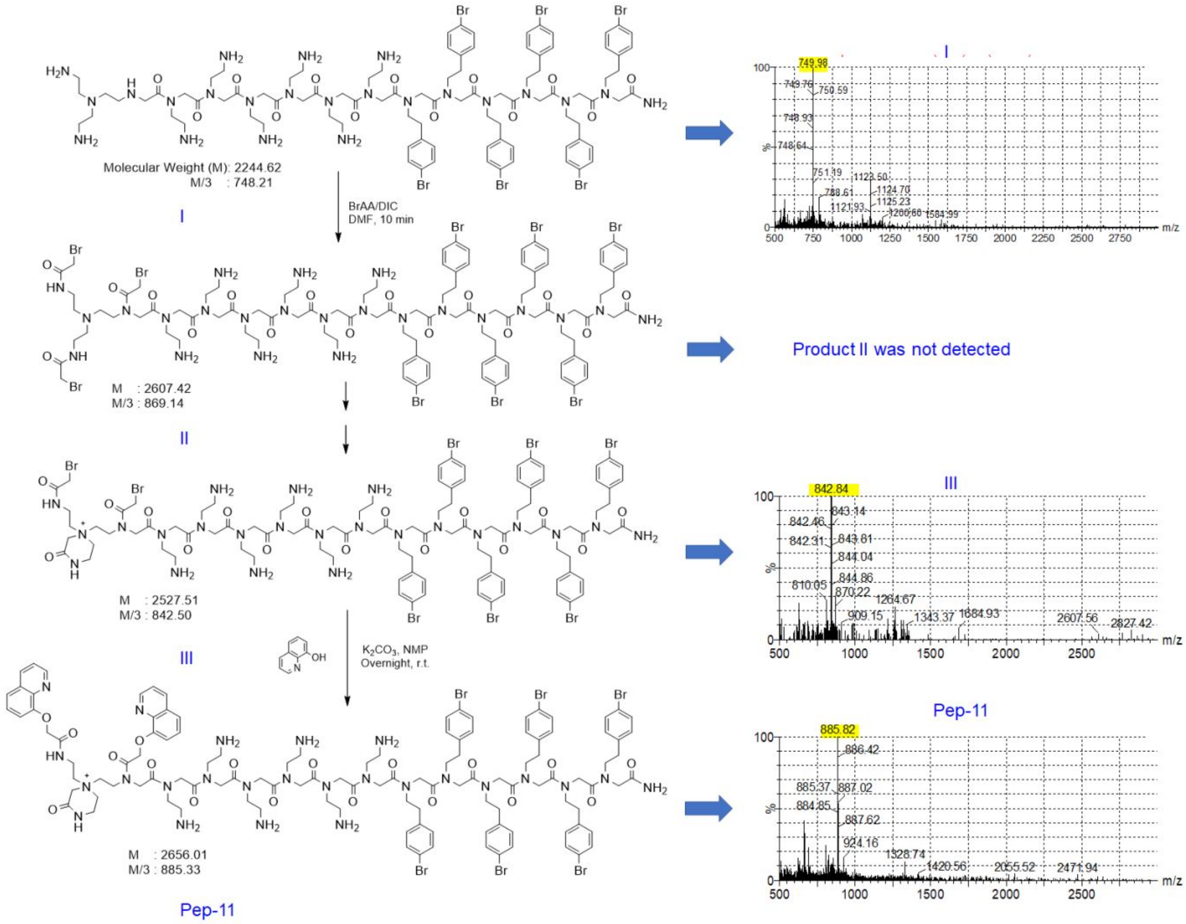

Figure S16. Representative reaction scheme for functionalizing of compound I to produce **Pep-11**. The LC-MS results demonstrate the formation of compound III, which contains a cyclic quaternary salt in the linker domain, instead of compound II during the acylation process. This finding provides evidence of an intramolecular reaction between bromo ester and tertiary amine of linker domain occurring during the reaction, leading to the unexpected formation of III rather than II. Compound III may likely be a mixture of two regio-isomers, as depicted in Figure S17. However, it was not possible to distinguish or separate the isomeric forms using LC-MS analysis.

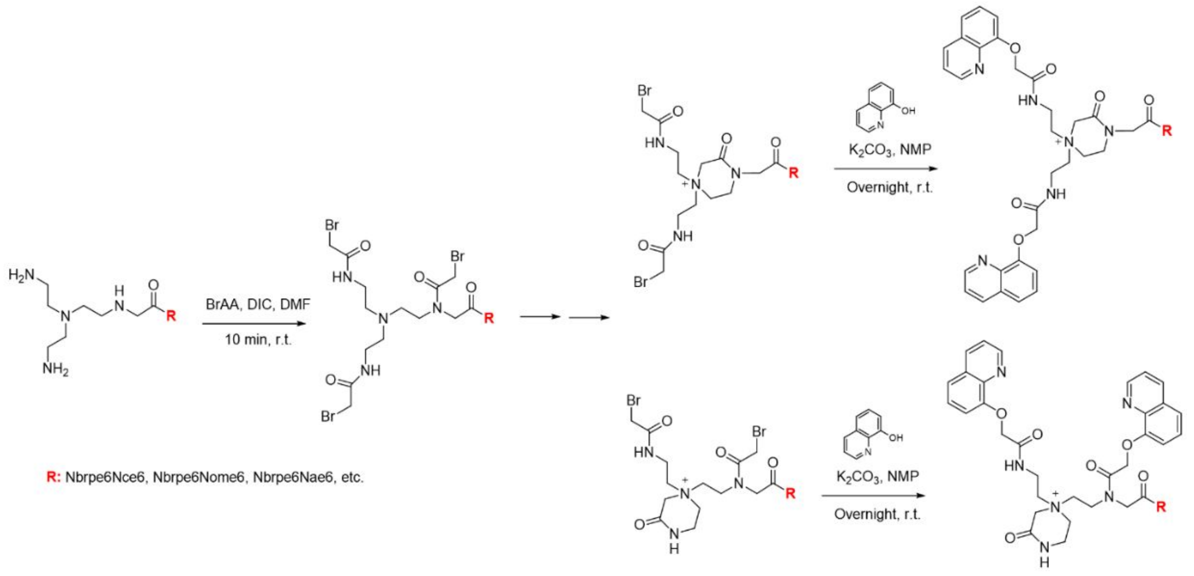

Figure S17. Proposed scheme for unexpected intramolecular cyclization reaction and possible products during the synthesis of membrane-forming peptoids containing two 8-hydroxyquinoline (Nqn) ligands.

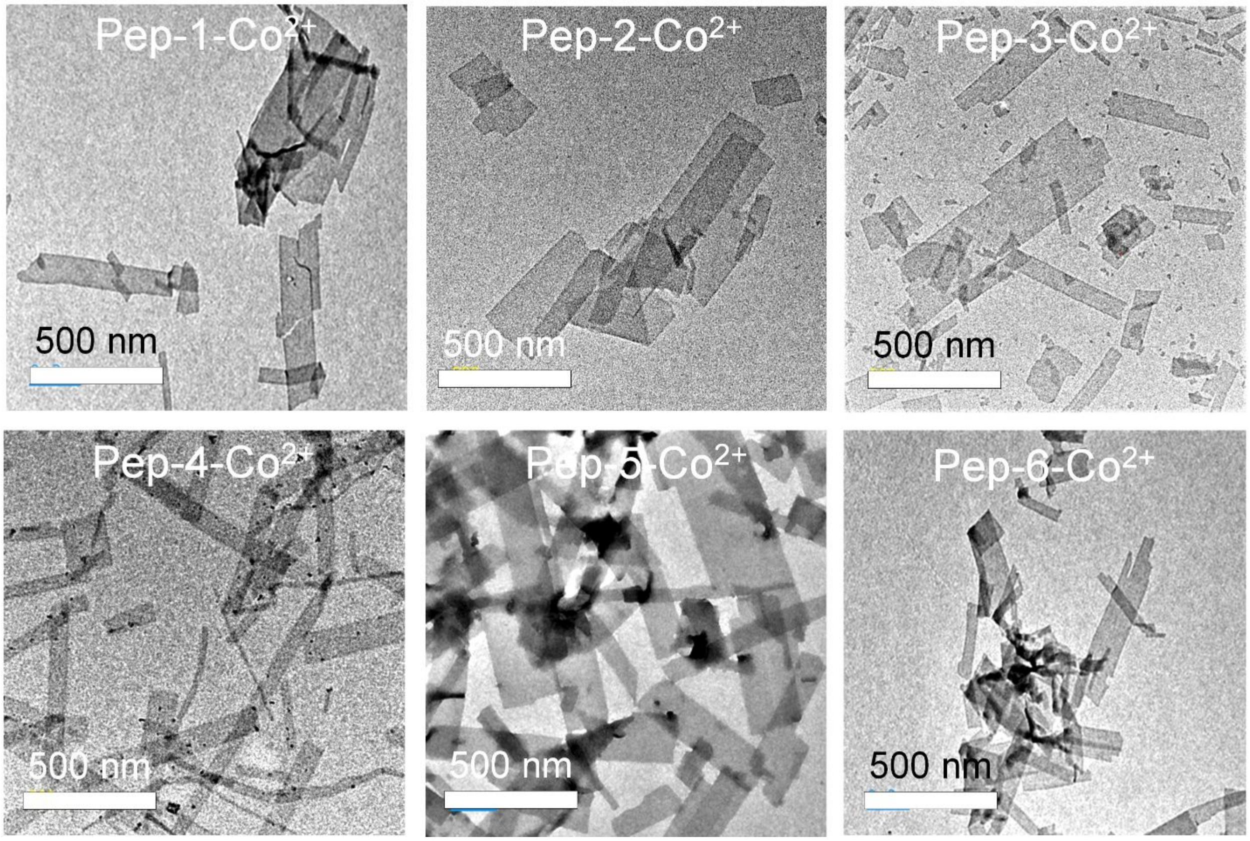

Figure S18. TEM images of Co^{2+}-containing membranes assembled from **Pep-1** to **Pep-6** respectively.

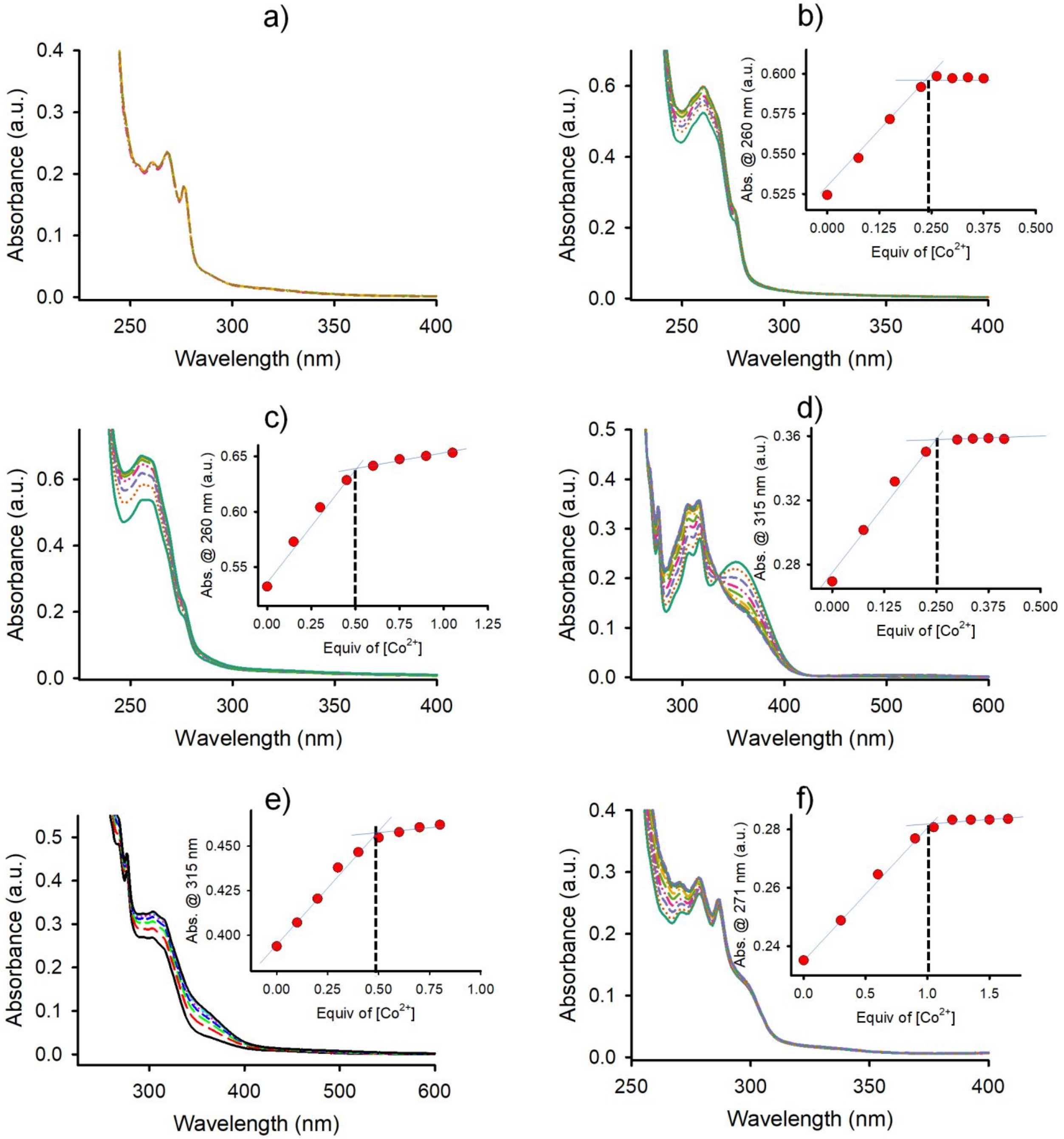

Figure S19. UV/ vis spectra and metal-to-peptoid ratio plots for the titration of different peptoids with Co^{2+} in acetonitrile. a) **Pep-1**. b) **Pep-2**. c) **Pep-3**. d) **Pep-4**. e) **Pep-5**. f) **Pep-6**.

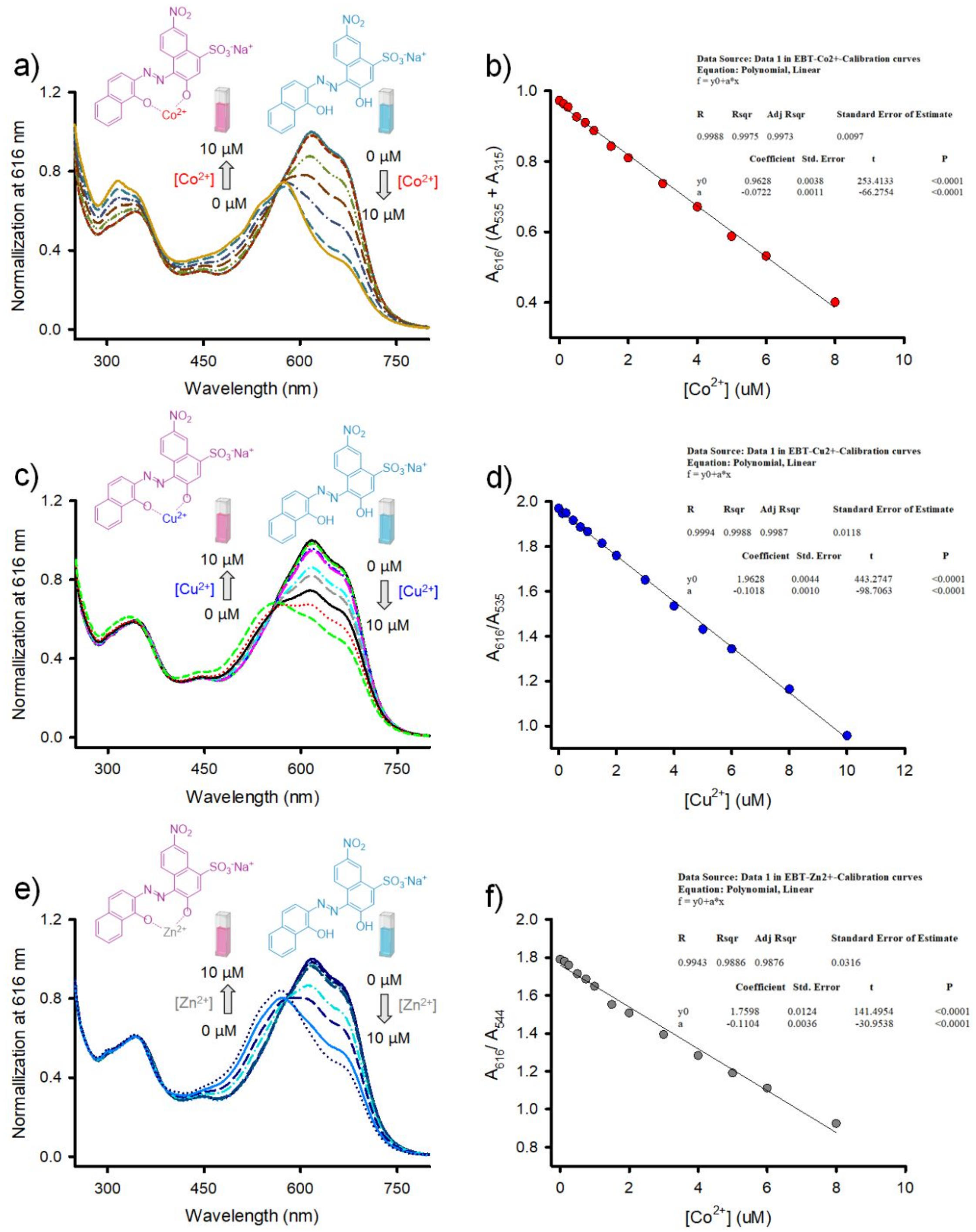

Figure S20. Changes in UV-vis spectra of Eriochrome Black T (EBT) dye as adding a given amount of (a) $Co(BF_4)_2$, (c) $Cu(BF_4)_2$ and (e) $Zn(BF_4)_2$. Calibartion curves for the presence of (b) $Co(BF_4)_2$, (d) $Cu(BF_4)_2$ and (f) $Zn(BF_4)_2$.

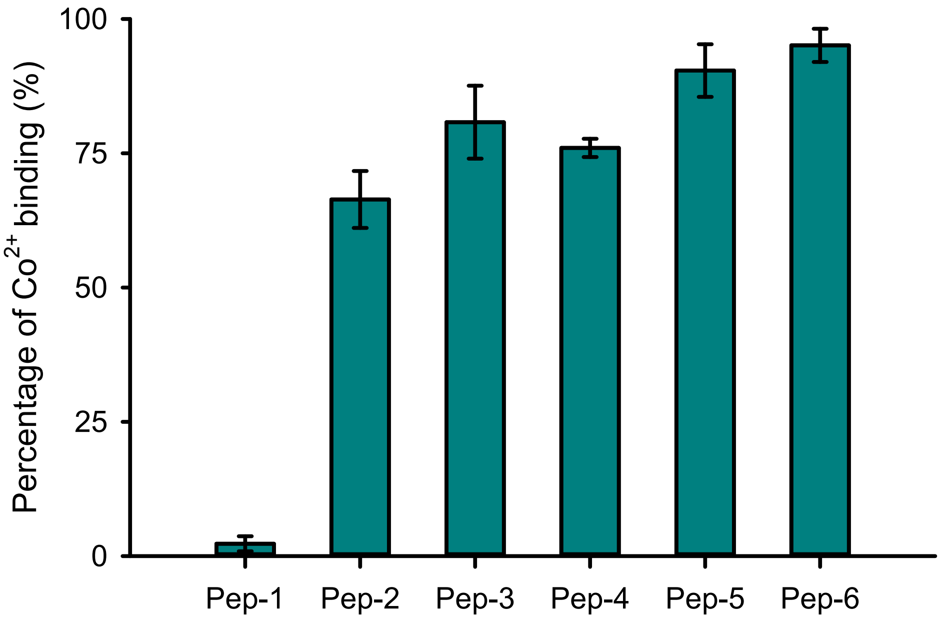

Figure S21. Percentage of Co^{2+} ions binding with peptoid chains within Co^{2+}-containing nanomembranes compared to their initial feeding amount. These nanomembranes were prepared by co-assmebling Co^{2+} and peptoids, using Co^{2+}: peptoid molar ratios of 1: 0.25 (for **Pep-2** and **Pep-4**), 1: 0.5 (for **Pep-3** and **Pep-5**), and 1:1 (for **Pep-6**).

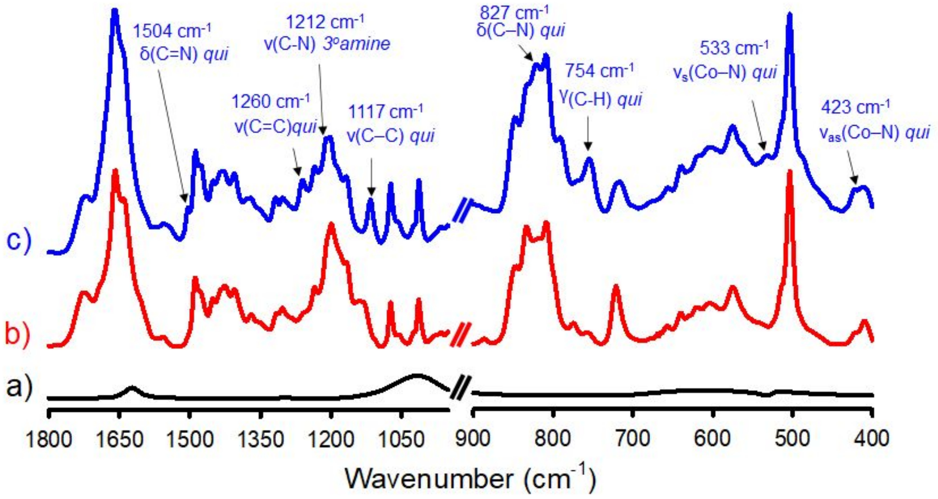

Figure S22. FT-IR analysis. a) $Co(BF_4)_2$. b) **Pep-5** nanomembrane. c) **Pep-5**-Co^{2+} nanomembrane.

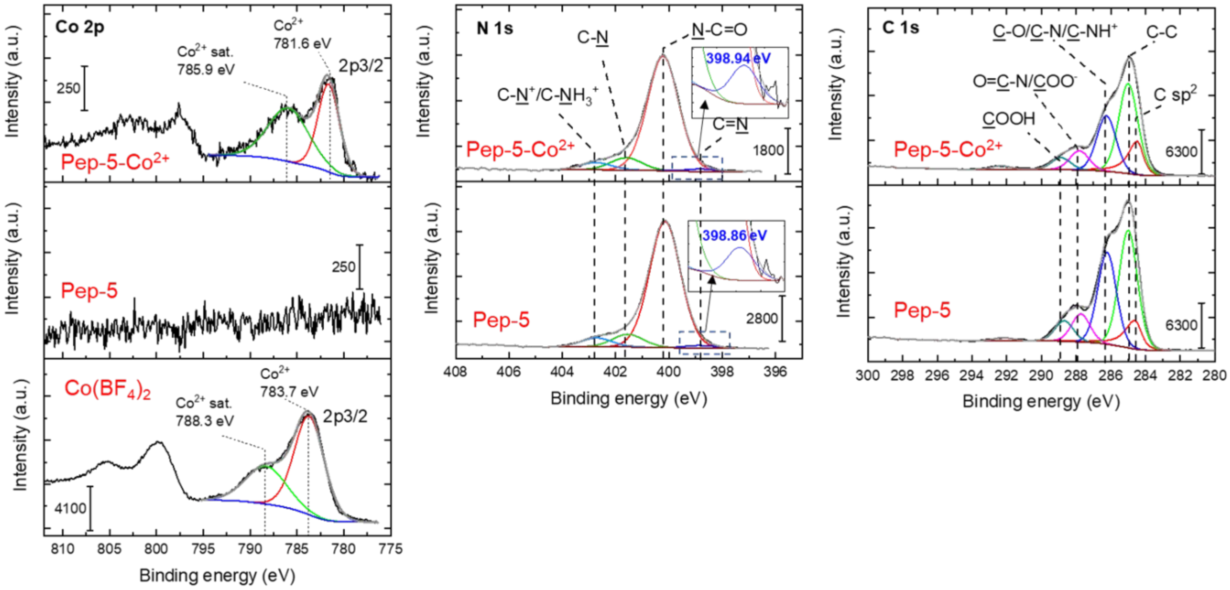

Figure S23. XPS analysis of **Pep-5**-Co^{2+}, **Pep-5** and $Co(BF_4)_2$. Co 2p confirmed the presence of Co^{2+} in **Pep-5**-Co^{2+}. In comparison with Co 2p3/2 signal of $Co(BF_4)_2$ precursor at 783.7 eV, the Co 2p3/2 from **Pep-5**-Co^{2+} sample shifted into lower binding energy (781.6 eV), suggesting changes in coordination environment of Co^{2+}. However, due to low concentration of Co^{2+} versus nitrogen and oxygen functional groups, changes in N 1s and C 1s are negligible.

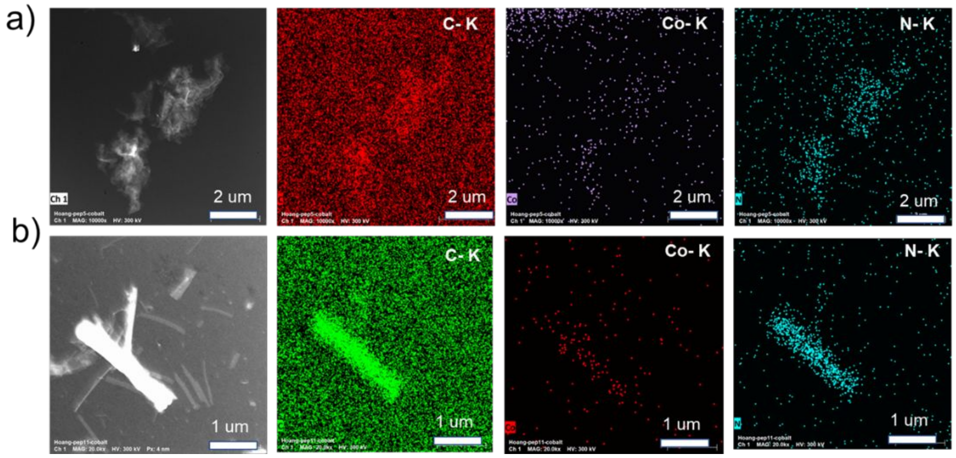

Figure S24. TEM/EDS analysis of metal-containing peptoid membranes. a) TEM/EDS data of **Pep-5**-Co^{2+} membranes. b) TEM/EDS data of **Pep-11**-Co^{2+} membrane.

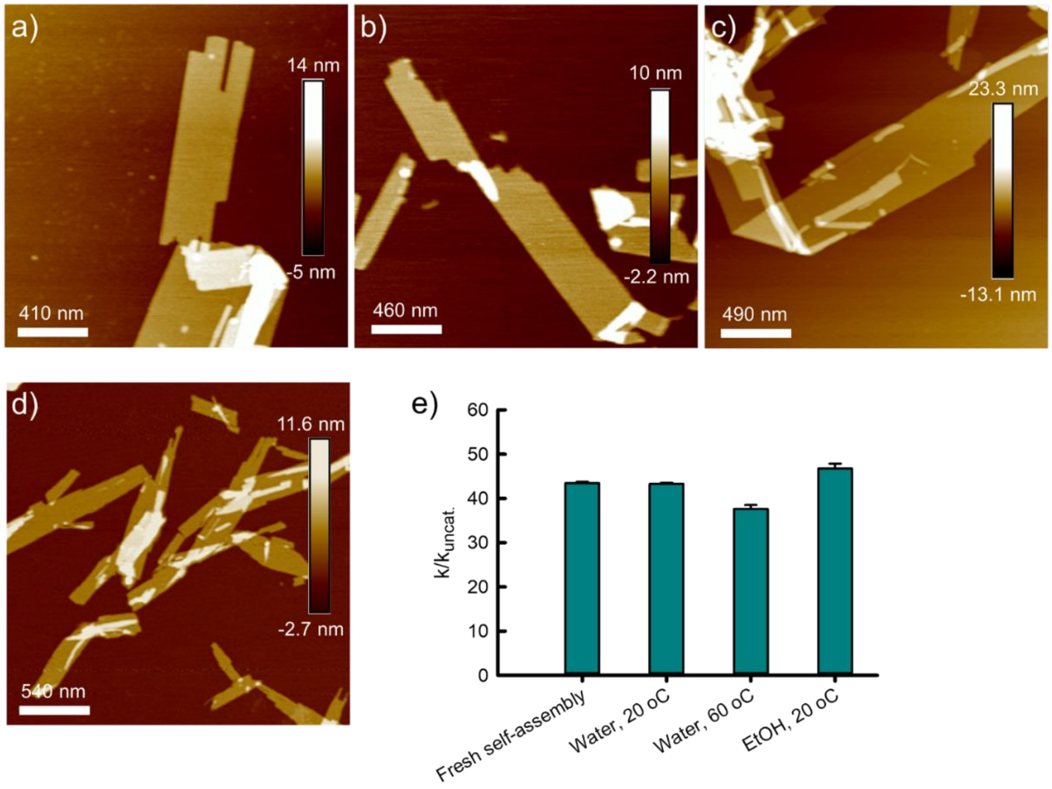

Figure S25. Stability of Co^{2+}-containing peptoid nanomembranes under various conditions. a) Heat at 60 °C for 2 h. b) Heat at 90 °C for 2h. c) In ethanol for 2h. d) In [NEM]= 20 mM, pH= 10 for1 week. e) Catalytic efficiency of **Pep-5**-Co^{2+} membranes under various conditions: freshly made; recovered from incubation in water at room temperature overnight, recovered from incubation in water at 60°C overnight; recovered from being incubated in ethanol at room temperature for 2 hours).

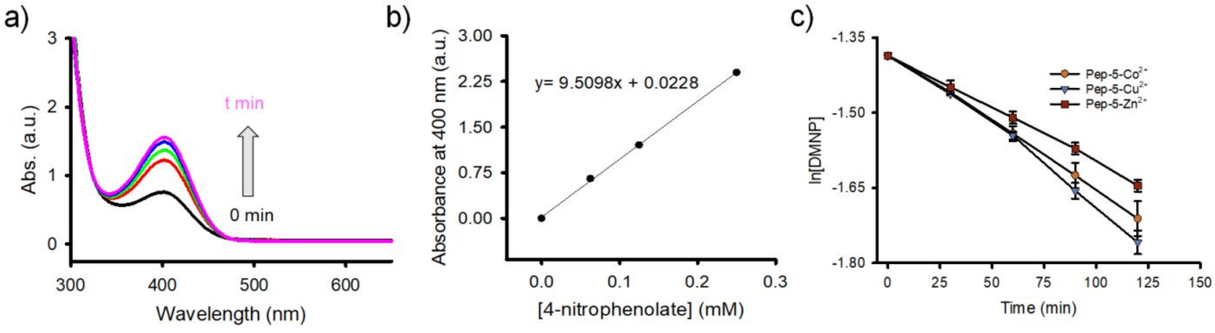

Figure S26. a) Representative changes in UV/vis spectra of DMNP hydrolysis reaction, b) Cablication curve of 4-nitrophenolate in [NEM] = 20 mM, pH= 10, and c) Effect of metal cations on the hydrolytic activity of metal-peptoid membranes.

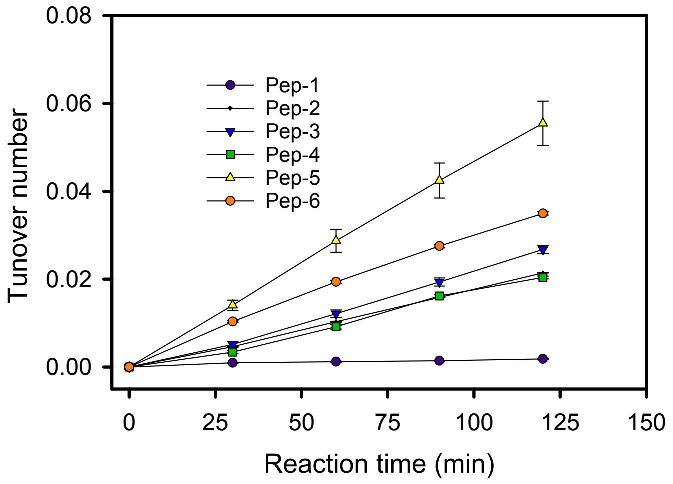

Figure S27. A plot of turnover number versus reaction time for different metalated peptoid membranes.

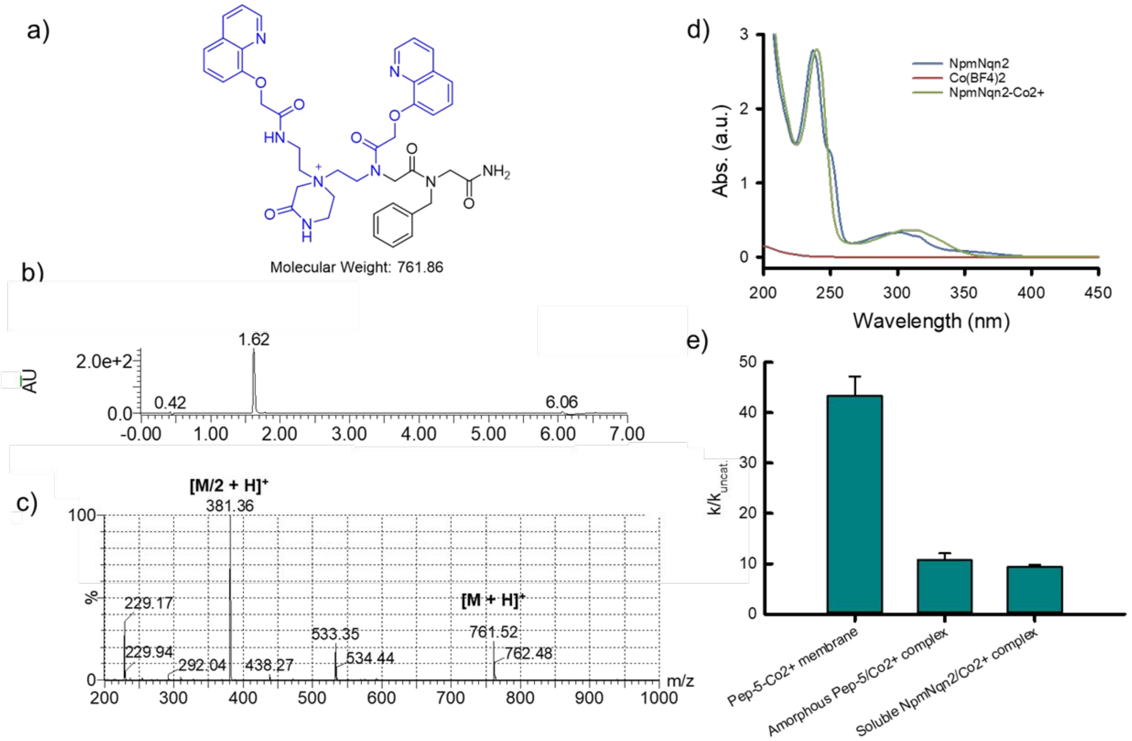

Figure S28. Characterization of **NpmNqn2** and the catalytic activity of its Co^{2+} complex. a) Chemical structure. b) LC-MS chromatogram with the gradient of 5 - 95% CH_3CN in H_2O. c) ESI^+ ionization pattern. d) UV/vis spectra of **NpmNqn2**, $Co(BF_4)_2$, and the **NpmNqn2**-Co^{2+} complex. Changes in the UV spectrum of the **NpmNqn2**-Co^{2+} complex, compared to **NpmNqn2**, confirm the presence of ligand and Co^{2+} coordination. e) Comparison of the activity of different catalytic systems in DMNP hydrolysis.

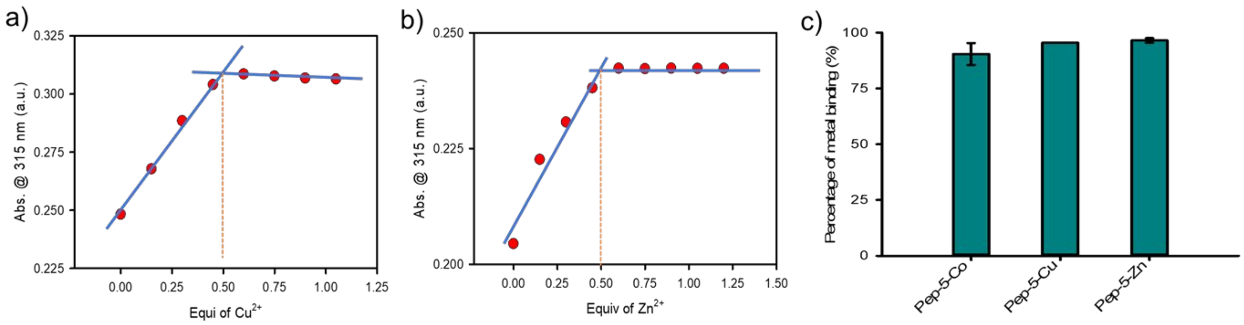

Figure S29. Kinetic of increase in absorbance of **Pep-5** upon adding given amounts of different metal cations. a) $Cu(BF_4)_2$. b) $Zn(BF_4)_2$. Quantitative amount of metals binding to **Pep-5** membranes (d).

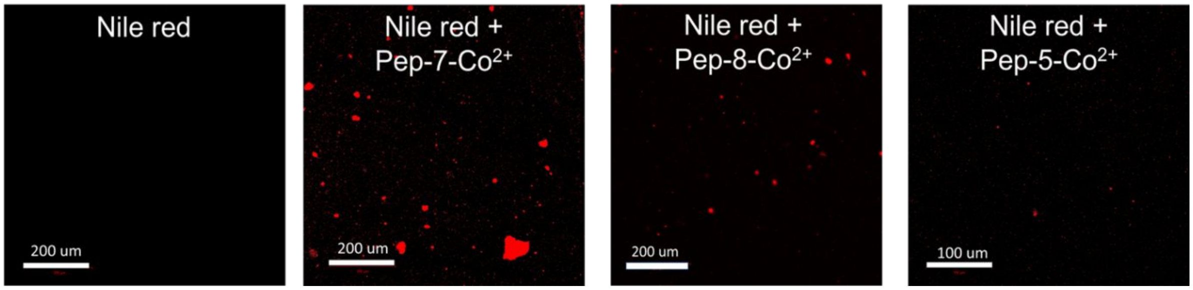

Figure S30. Fluorescent microscopy images of Nile red and Nile red/Co^{2+}-containing peptoid membranes. Observation of red clusters, resulted from the interaction of Nile red and hydrophobic regions of the peptoid structure, provides direct evidence of the presence of aggregated regions within certain peptoid assemblies.

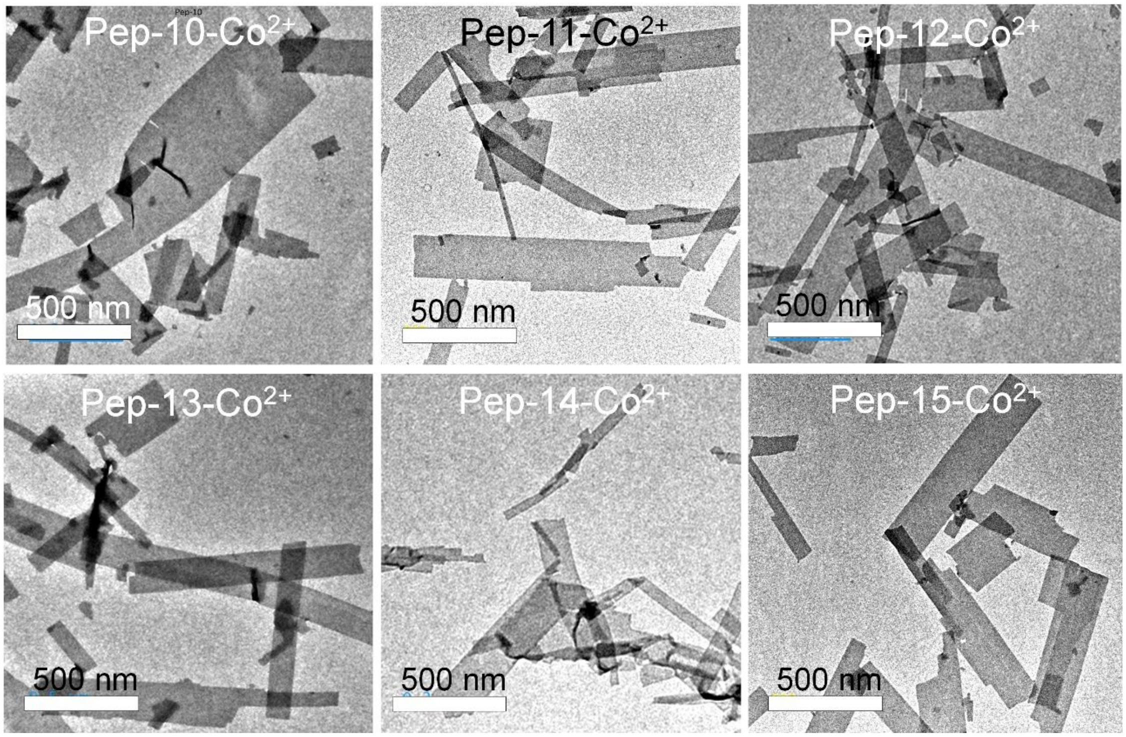

Figure S31. TEM images of Co^{2+}-containing membranes assembled from **Pep-10** to **Pep-15** respectively.

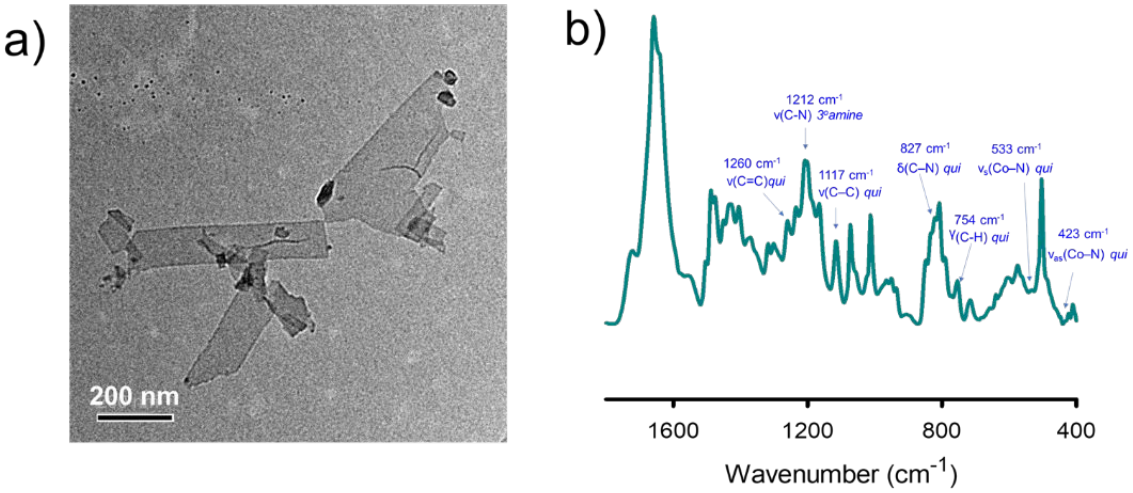

Figure S32. Characterizations of resuablity of **Pep-5**-Co^{2+} membranes. a) TEM image shows that **Pep-5**-Co^{2+} membranes are still present in well-defined shapes after three-cycle hydrolytic reactions. b) FT-IR spectrum of **Pep-5**-Co^{2+} membranes after three-cycle hydrolysis experiments. The characteristic bands at 533 and 423 cm^{-1} correspond to υ (Co–N), confirming the presence of Nqn-Co^{2+} active sites.[6]

Table S1. A summary of DMNP hydrolysis activities triggered in NEM buffer under various conditions ([DMNP] = 0.25 mM and [NEM]= 20 mM, pH10).

Entry	Catalyst	pH	TOF[a] ($\times 10^{-7}$ min^{-1})
1	-	10	-
2	**Pep-1**-Co^{2+} membrane	10	1.2
3	**Pep-2**-Co^{2+} membrane	10	89
4	**Pep-3**-Co^{2+} membrane	10	120
5	**Pep-4**-Co^{2+} membrane	10	70
6	**Pep-5**-Co^{2+} membrane	10	690
7	**Pep-6**-Co^{2+} membrane	10	310
8	Amorphous **Pep-5**/Co^{2+}	10	1.6
9[b]	Amorphous **Pep-5**/Co^{2+} complex	10	15
10[c]	Soluble **NpmHqn2**/Co^{2+} complex	10	29
11[d]	**Pep-5** membrane/ Co^{2+}	10	60
12	**Pep-5**-Cu^{2+} membrane	10	722
13	**Pep-5**-Zn^{2+} membrane	10	451
14	**Pep-7**-Co^{2+} membrane	10	38
15	**Pep-8**-Co^{2+} membrane	10	79
16	**Pep-9**-Co^{2+} membrane	10	260
17	**Pep-10**-Co^{2+} membrane	10	280
18	**Pep-11**-Co^{2+} membrane	10	5,000
19	**Pep-12**-Co^{2+} membrane	10	140
20	**Pep-13**-Co^{2+} membrane	10	930
21	**Pep-14**-Co^{2+} membrane	10	14,100
22	**Pep-15**-Co^{2+} membrane	10	5,700
23	**Pep-11**-Co^{2+} membrane	9	3,100
24	**Pep-11**-Co^{2+} membrane	8	1,500
25	**Pep-11**-Co^{2+} membrane, 60 ^{o}C	10	14,670
26	**Pep-5-Pep-1**-Co^{2+} (1:2) membrane	10	579
27	**Pep-5-Pep-1**-Co^{2+} (1:1) membrane	10	1,270
28	**Pep-5-Pep-1**-Co^{2+} (2:1) membrane	10	591
29[e]	**Pep-5**-Co^{2+} membrane, 2nd cycle	10	669
30[f]	**Pep-5**-Co^{2+} membrane, 3rd cycle	10	586
31[g]	**Pep-5**-Co^{2+} membrane, 4th cycle	10	589
32[k]	**Pep-5**-Co^{2+} membrane, 5th cycle	10	510
33[l]	**Pep-5**-Co^{2+} membrane, 6th cycle	10	503
34[m]	**Pep-5**-Co^{2+} membrane, 7th cycle	10	499

[a]Turnover frequency determined at 60 min.

[b]A mixture of amorphous **Pep-5** and $Co(BF_4)_2$ premixed for a week.

[c]A mixture of soluble NpmNqn2 and Co(BF4)2 premixed for a week.

[d]A mixture of **Pep-5** membrane and $Co(BF_4)_2$ premixed for a week.

[e–m]Co^{2+}-containing membranes were recovered following specific protocol reported in SI.

Table S2. The Michaelis-Menten kinetics toward DMNP hydrolysis by various catalysts

Catalysts	Condition	Activity	Ref
PTE-Co^{2+}	60°C, pH = 9.0,<br>100 mM CHES	Complete inactivation	[7]
**Pep-11**-Co^{2+} membrane	60°C, pH = 10.0,<br>20 mM NEM	$K_{cat}/K_m = 2.45\ M^{-1} s^{-1}$<br>$K_{cat} = 468 \times 10^{-6}\ s^{-1}$	This work

References

1. Jin, H.; Jiao, F.; Daily, M. D.; Chen, Y.; Yan, F.; Ding, Y.-H.; Zhang, X.; Robertson, E. J.; Baer, M. D.; Chen, C.-L., Highly stable and self-repairing membrane-mimetic 2D nanomaterials assembled from lipid-like peptoids. *Nat. Commun.* **2016,** *7* (1), 12252.
2. Wang, M.; Song, Y.; Zhang, S.; Zhang, X.; Cai, X.; Lin, Y.; Yoreo, J. J. D.; Chen, C.-L., Programmable two-dimensional nanocrystals assembled from POSS-containing peptoids as efficient artificial light-harvesting systems. *Sci. Adv.* **2021,** *7* (20), eabg1448.
3. Yilmaz, M. D.; Oktem, H. A., Eriochrome Black T–Eu3+ Complex as a Ratiometric Colorimetric and Fluorescent Probe for the Detection of Dipicolinic Acid, a Biomarker of Bacterial Spores. *Anal. Chem.* **2018,** *90* (6), 4221-4225.
4. Dubenskaya, L. O.; Levitskaya, G. D., Use of Eriochrome Black T for the polarographic determination of rare earth metals. *J. Anal. Chem.* **1999,** *54* (7), 742-744.
5. Tran, H.; Gael, S. L.; Connolly, M. D.; Zuckermann, R. N., Solid-phase submonomer synthesis of peptoid polymers and their self-assembly into highly-ordered nanosheets. *J. Vis. Exp.* **2011,** (57), e3373.
6. Nayak, S. C.; Das, P. K.; Sahoo, K. K., Synthesis and characterization of some cobalt(III) complexes containing heterocyclic nitrogen donor ligands. *Chem. Papers* **2003,** *57* (2), 91-96.
7. Rochu, D.; Viguié, N.; Renault, F.; Crouzier, D.; Froment, M. T.; Masson, P., Contribution of the active-site metal cation to the catalytic activity and to the conformational stability of phosphotriesterase: temperature- and pH-dependence. *Biochem. J.* **2004,** *380* (Pt 3), 627-33.
